# Noise‐induced reduction and early recovery of superior paraolivary nucleus sound‐offset responses

**DOI:** 10.1113/JP289987

**Published:** 2026-04-16

**Authors:** Mihai Stancu, Ezhilarasan Rajaram, Joseph A. Kroeger, Benedikt Grothe, Conny Kopp‐Scheinpflug

**Affiliations:** ^1^ Faculty of Biology, Division of Neurobiology Ludwig‐Maximilians‐University Munich Germany; ^2^ Graduate School of Systemic Neurosciences Ludwig‐Maximilians‐University Munich Germany; ^3^ Munich Cluster for Systems Neurology (SyNergy) Munich Germany

**Keywords:** central gain, inhibitory synaptic transmission, intrinsic excitability, noise‐induced hearing loss, sound offset responses, spontaneous activity, superior paraolivary nucleus

## Abstract

**Abstract:**

Neural circuits exhibit remarkable plasticity in response to varying intensities of sensory input. The temporal dynamics and cellular mechanisms underlying this plasticity are highly heterogeneous and possibly specific to individual circuits. Excessive noise exposure causes damage of peripheral auditory structures, such as cochlear hair cells and auditory nerve fibres, reducing afferent projection to downstream structures and initiating cascades of long‐lasting compensatory changes in central auditory circuits. Amongst these changes, increased neuronal excitability, elevated spontaneous firing and increased neural gain were reported across multiple structures between the cochlear nucleus and auditory cortex. However, these findings primarily involved neurons that were responsive to sound onset (ON) and driven by excitation. Much less is known about the impact of noise exposure on neurons that are selectively activated by sound offset (OFF) and are driven by inhibition. We addressed this gap in knowledge by investigating the effects of noise exposure on intrinsic membrane properties, synaptic input patterns and sound‐evoked activity in superior paraolivary nucleus (SPN) neurons, which are a population of neurons specialized for encoding sound offset. Immediately after noise exposure, SPN neurons were unresponsive to sound offset. Within the next 24 h, we observed a significant increase in the number of inhibitory synaptic terminals impinging upon SPN neurons, which was corroborated by elevated frequencies and amplitudes of inhibitory postsynaptic currents. At the same time, SPN neurons exhibited higher intrinsic excitability. Together, these changes encouraged recovery of sound‐evoked OFF responses 24 h following noise exposure, suggesting circuit‐specific compensatory mechanisms that enable sound OFF encoding soon after peripheral auditory insult.

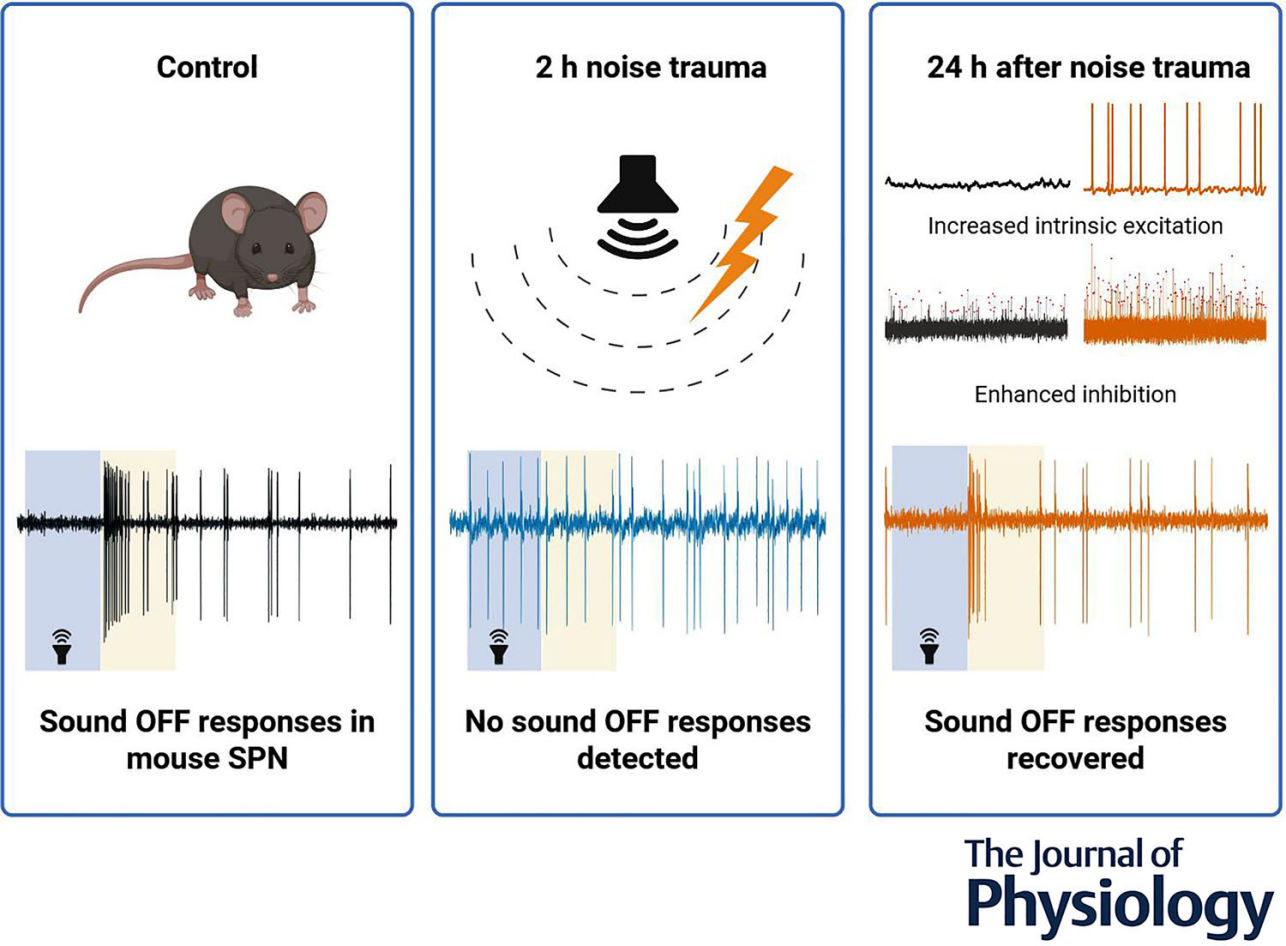

**Key points:**

- Sound‐offset (OFF) responses mark the critical temporal boundary when a sound terminates; this enables encoding of sound duration and the detection of gaps in sounds and calls.
- In the mouse model, OFF responses are generated *de novo* in the superior paraolivary nucleus by combining sound‐evoked inhibitory input with the intrinsic membrane properties of the neurons.
- The impact of noise over‐exposure on these OFF responses and its implications for subsequent auditory processing is not well understood.
- Combining patch‐clamp recording, immunohistochemistry and *in vivo* electrophysiology, we show that superior paraolivary nucleus neurons exhibit increased excitability and enhanced inhibition following noise over‐exposure.
- These compensatory changes help to mediate early recovery of sound OFF responses to loud stimuli, despite the loss of auditory sensitivity at lower sound intensities.

## Graphical abstract

**Abstract figure legend** Acoustic over‐exposure transiently disrupts auditory temporal processing in mouse superior paraolivary nucleus neurons. In control conditions, neurons exhibit robust sound‐offset (OFF) responses, which are abolished immediately following noise trauma, indicating impaired temporal encoding. Recovery by 24 h post‐trauma is supported by adaptive changes, including increased intrinsic excitability and, notably, enhanced inhibition; a finding that contrasts with prevailing expectations in the field. This rapid restoration of OFF responses suggests strong circuit resilience and points to a potential behavioural importance of offset signalling.

## Introduction

Auditory neurons in the brain are highly specialized for continuous adaptation to acoustic stimuli, enabling dynamic encoding and processing of the temporal structure of sound. In addition to neuronal populations that respond to sound onset (ON) or sustained acoustic features, distinct neural populations are selectively responsive to sound offset (OFF) (Kopp‐Scheinpflug et al., 2018). Encoding temporal discontinuities arising from transient changes in sound intensity, such as those occurring during vocal communication via both ON and OFF responses, is thought to effectively increase the sampling rate, thereby enhancing the precision of temporal auditory processing. Deficits in temporal auditory processing are an often‐overlooked consequence of noise exposure. Although exposure to traumatic noise is well known to elevate auditory thresholds and thereby reduce sensitivity to low‐intensity sounds, impaired processing of suprathreshold temporal cues can compromise speech perception, particularly in noisy environments (Radziwon et al., 2019). These deficits in temporal processing raise the question of whether and how neuronal populations encoding sound OFF are affected by noise exposure.

Reductions in afferent input following noise exposure induce compensatory plasticity within the central auditory system, such as an upregulation of central auditory responsiveness, a phenomenon commonly referred to as enhanced central gain (Auerbach et al., 2014; Chambers et al., 2016; McGill et al., 2022; Schrode et al., 2018). A seminal review paper has proposed three principal mechanisms underlying central gain enhancement: (1) reduced inhibitory synaptic transmission; (2) increased excitatory synaptic transmission; and (3) alterations in intrinsic neuronal excitability (Auerbach et al., 2014). The nature and extent of these adaptations are influenced by the intensity and duration of noise exposure, the postexposure time point of analysis and the specific auditory brain region under investigation.

Noise exposure can be classified broadly into high‐intensity exposures, which cause auditory threshold shifts of >50 dB and are typically associated with hidden hearing loss (Furman et al., 2013; Kujawa & Liberman, 2009), and moderate exposures, which induce threshold shifts of ≤40 dB that often recover within 24 h (Syka & Rybalko, 2000). Rapid adaptive mechanisms emerge within minutes of exposure and are aimed at restoring neural responsiveness by reducing inhibition (Balaram et al., 2019; Sarro et al., 2008; Schrode et al., 2018; Sturm et al., 2017). These mechanisms can overshoot, resulting in hyperexcitability throughout the central auditory pathway (Hockley et al., 2020; Li et al., 2013, 2015; Pilati, Ison et al., 2012; Pilati, Large et al., 2012; Wu, Martel et al., 2016; Yang et al., 2012). Consequently, spontaneous and stimulus‐evoked firing rates of auditory neurons, from the cochlear nucleus to the auditory cortex, are typically elevated following noise exposure (Auerbach et al., 2014; Radziwon et al., 2019).

To date, most studies investigating cellular plasticity following noise exposure have focused on neurons responsive to sound onset or sustained sound features. In contrast, the effects of noise exposure on neurons selective for sound offset remain largely unexplored. To address this gap in knowledge, we examined how sound‐offset‐responsive neurons in the superior paraolivary nucleus (SPN) are affected by noise exposure. The SPN is an ideal model for investigating noise‐induced plasticity in inhibitory circuits, because here the inhibitory input, which elsewhere merely modulates neural responses, single‐handedly generates bursts of action potentials at sound offset (Felix et al., 2011; Grothe, 1994; Kopp‐Scheinpflug et al., 2011; Rajaram et al., 2019, 2020).

We used a combination of immunohistochemistry, *in vitro* whole‐cell patch‐clamp recordings from acute brain slices and *in vivo* single‐unit recordings to assess the effect of noise exposure on mechanisms underlying OFF response generation in SPN neurons. The noise exposure protocol was chosen to induce threshold shifts of ≤40 dB, and neural responses were assessed at two time points: immediately after exposure (0 h) and 24 h postexposure. Consistent with previous reports, we observed increased intrinsic neuronal excitability following noise exposure. However, contrary to the prevailing hypothesis of reduced inhibition as a driver of central gain, we found that inhibitory input to SPN neurons was not diminished but rather enhanced. This observation was associated with the recovery of sound OFF responses, including spectral and temporal tuning properties comparable to control animals.

### Methods

All experimental procedures were reviewed and approved by the Bavarian district government (TVV AZ: 55.2‐2532.Vet_02‐18‐118) and were carried out according to the European Communities Council Directive (2010/63/EU). C57Bl6J mice originating from Charles River Laboratories were bred and housed in a vivarium at the Ludwig‐Maximilians‐University Biocenter with a normal light–dark cycle (12 h–12 h light–dark) and with food and water *ad libitum*. Mice of both sexes, aged 26 ± 5 days, were used for the physiological and histological experiments. This postweaning age was selected to ensure relative maturation of SPN neurons while also permitting high yield and quality in patch‐clamp recordings (Rajaram et al., 2020).

#### Noise exposure

Bandpass‐filtered noise between 8 and 16 kHz was generated using RPvdsEx (Tucker Davis Technologies) and presented via earphones (MF1 Magnetic Speakers, Tucker‐Davis Technologies) for 2 h at a level of 107 dB SPL. During the noise exposure, animals were anaesthetized with an i.p. injection of MMF (medetomidin 0.5 mg/kg, midazolam 5 mg/kg and fentanyl 0.05 mg/kg) and placed on a temperature‐controlled heating pad (ATC1000, WPI) in a soundproof chamber (Industrial Acoustics, Niederkruchten, Germany). Auditory brainstem responses (ABRs) were recorded before noise exposure (control), immediately after noise exposure (0 h) to test for the efficacy of the exposure and also after 24 h. After the ABRs, animals were either killed to prepare brain slices for the subsequent patch‐clamp experiments and immunolabelling, or transferred back into their cage to be used at the 24 h time point.

#### Auditory brainstem response recordings

ABRs were recorded in mice anaesthetized with an i.p. injection of MMF (see previous subsection). During the experiments, mice were placed on a temperature‐controlled heating pad (ATC1000, WPI, Sarasota, FL, USA) in a soundproof chamber (Industrial Acoustics, Niederkruchten, Germany). Disposable needle electrodes (Rochester Electro‐Medical, Inc., Coral Springs, FL, USA) were placed subdermally at the vertex of the head (reference), ventral to the pinna (active) and near the base of the tail (earth). Electrodes were attached to a low‐impedance head stage (RA4LI, Tucker Davis Technologies, Alachua, FL, USA) and connected to the auditory processor (RZ6, Tucker Davis Technologies) with an optical input cable through a preamplifier (RA16PA, Tucker Davis Technologies; amplification factor of 250). Custom software (Brandon Warren, University of Washington) was used to address the speakers *in situ* (MF1, Tucker Davis Technologies), generate stimuli and record ABR waveforms. Stimuli consisted of broadband clicks (100 µs duration) presented 1000 times at varying intensities (0–90 dB SPL, 1–10 dB steps). Responses were averaged across 1000 stimulus repetitions, and thresholds were determined as the sound intensity at which at least two peaks could be distinguished in the average ABR waveform. If no threshold was detected at 90 dB SPL, a value of 90 dB SPL was given as the threshold.

#### Immunohistochemistry and imaging

Mice received an overdose of pentobarbitone (400 mg/kg body weight; i.p.) and were perfusion fixed with 4% paraformaldehyde intracardially. Following overnight postfixation in 4% paraformaldehyde, brainstems were sectioned coronally at 50 µm thickness using a vibrating blade microtome (V1200S, Leica, Wetzlar, Germany). Slices were washed three times for 10 min in PBS before transferring them to a blocking solution containing 1% bovine serum albumin, 0.5% Triton X‐100 and 0.1% saponin in PBS. Primary antibodies against the glycine transporter type 2 (GlyT2; Millipore no. 1773; 1:1000) stained glycinergic terminals. Anti‐gephyrin antibodies (SySy no. 147021; 1:300) were used to visualize the location of postsynaptic glycine receptors. Neuronal cell bodies were labelled with MAP2 (chicken anti‐MAP2; Neuromics, CH22103; 1:1000). Incubation with primary antibodies took place for 24 h at 4°C. Tissue was again washed three times for 10 min in PBS at room temperature before incubation for 24 h at 4°C with secondary antibodies diluted in blocking solution. Then, sections were rinsed three times for 10 min in PBS and coverslipped with Vectashield mounting medium. Confocal optical sections were acquired with a confocal laser‐scanning microscope equipped with HCX PL APO CS ×20/NA 0.7 and HCX PL APO Lambda Blue ×63/NA 1.4 immersion oil objectives (Leica). Fluorochromes were visualized with excitation wavelengths of 405 nm (emission filter, 410–430 nm) for amino‐methylcoumarin, 488 nm (emission filter, 510–540 nm) for Alexa 488, and 561 nm (emission filter, 565–585 nm) for Cy3. For each optical section, the images were collected sequentially for the different fluorochromes. Stacks of eight‐bit greyscale images were obtained with axial distances of 290 nm between optical sections and pixel sizes of 120–1520 nm depending on the selected zoom factor and objective. To improve the signal‐to‐noise ratio, images were averaged from three successive scans. Red, green, blue (RGB) stacks, montages of RGB optical sections, and maximum‐intensity projections were assembled using the ImageJ StackGroom plugin.

#### *In vitro* electrophysiology

Mice were briefly anaesthetized with isoflurane and rapidly killed by decapitation. Coronal brainstem sections (150–200 µm thick) containing the SPN (Rajaram et al., 2020) were cut in an ice‐cold high‐sucrose, low‐sodium artificial cerebral spinal fluid (ACSF). Brainstem slices were maintained after slicing in normal ACSF at 37°C for 30–45 min, after which they were stored in a slice maintenance chamber at room temperature (∼22°C) until being transferred into the recording chamber. The composition of the normal ACSF was as follows (mM): 125 NaCl, 2.5 KCl, 26 NaHCO_3_, 10 glucose, 1.25 NaH_2_PO_4_, 2 sodium pyruvate, 3 myoinositol, 2 CaCl_2_, 1 MgCl_2_ and 0.5 ascorbic acid. pH was adjusted to 7.4, bubbled with 95% O_2_–5% CO_2_. For the low‐sodium ACSF, CaCl_2_ and MgCl_2_ concentrations were 0.1 and 4 mM, respectively, and NaCl was replaced by 200 mM sucrose. Experiments were conducted at 36 ± 1°C, maintained by an inline feedback temperature controller and heated stage (Warner Instruments). The recording chamber was continuously perfused with ACSF at a rate of 1–2 ml/min. Whole‐cell patch‐clamp recordings were made from visually identified SPN neurons using an EPC10/2 HEKA amplifier (HEKA Electronik), sampled at 50 kHz and filtered at 10 kHz. Patch pipettes were pulled from borosilicate glass capillaries (Warner Instruments) using a DMZ Universal electrode puller (Zeitz‐Instuments Vertriebs GmbH), filled with a patch solution containing the following (mM): 126 potassium gluconate, 4 KCl, 40 HEPES, 5 EGTA, 1 MgCl_2_, 5 disodium phosphocreatine, 0.2% biocytin and 292 mosmol/L. HEPES at 40 mM supports a robust pH buffering of the neurons. pH was adjusted to 7.2 with KOH. Data were corrected for liquid junction potentials of −13.8 mV. Electrode resistance was between 2.4 and 6 MΩ. Synaptic responses were evoked by stimulation of afferent fibres with concentric bipolar electrodes (FHC Inc., no. CBARC75). Voltage pulses were generated by the HEKA amplifier and postamplified by an isolated pulse stimulator (AM Systems). Inhibitory currents were recorded in ACSF containing 6,7‐dinitroquinoxaline‐2,3‐dione (10 µM) and d‐2‐amino‐5‐phosphonopentanoic acid (50 µM) to block AMPA and NMDA glutamate receptors, respectively. Miniature inhibitory postsynaptic currents (mIPSCs) were recorded in the presence of 1 µM tetrodotoxin, and for each cell data were collected over 60 s (three repetitions of 10 × 2 s sweeps). Pharmacological compounds were obtained from Sigma‐Aldrich.

#### *In vivo* single‐cell recording

Mice were anaesthetized with an i.p. injection of MMF (see above) and placed on a temperature‐controlled heating pad in a soundproof chamber (see above). The depth of anaesthesia was monitored using the toe withdrawal reflex, and animals responding were given supplemental MMF at one‐third of the initial dose. The mice were stabilized in a custom stereotaxic device. An incision was made at the top of the skull, followed by a craniotomy immediately anterior to the lambda suture intersection. The skull was tilted to provide access to the auditory brainstem. The SPN is located between 4600 and 4850 µm posterior and between 800 and 1150 µm lateral to bregma at an average depth of 5500–6100 µm. An earth electrode was placed in the muscle at the base of the neck. Glass microelectrodes were pulled from glass capillaries such that the resistance was 5–20 MΩ when filled with 3 M KCl solution. Signals were amplified (AM Systems, Neuroprobe 1600), filtered (300–3000 Hz; Tucker‐Davis‐Technologies PC1) and recorded (∼50 kHz sampling rate) with a Fireface UFX audio interface (RME). AudioSpike software (HoerrTech) was used to calibrate the multi‐field magnetic speakers, generate stimuli and record action potentials. Sound calibration was performed within the same software environment used for the experiments [AudioSpike, the free software tool for electrophysiological measurements using audio interfaces (v.2.1.0.0), Zenodo, https://doi.org/10.5281/zenodo.13847820]. The calibration set‐up included a Type 4939‐A‐011 free‐field microphone (Brüel & Kjær, Denmark) connected to a Type 2690 amplifier (Brüel & Kjær), with a Type 4231 sound calibrator (Brüel & Kjær) providing a reference tone. Acoustic stimuli were delivered via two MF‐1 speakers (Tucker‐Davis Technologies, USA) driven by an RB‐976MkII Rotel amplifier (Rotel, Japan). Signals were acquired through a Fireface UFX audio interface (RME Audio, Germany) and processed in AudioSpike. Stimuli consisted of pure tones (100 ms duration, 5 ms rise/fall time) at varying intensity (0–90 dB SPL) and were presented through hollow ear bars connected to the speakers. Post‐stimulus time histograms (PSTHs) were assessed at the characteristic frequency (CF) at different sound intensities. At the end of the *in vivo* recording sessions, animals were killed by an overdose of pentobarbitone (400 mg/kg body weight; i.p.), then perfusion‐fixed with 4% paraformaldehyde for subsequent histological analyses.

#### Experimental design and statistical analysis

We report *n* as the number of neurons recorded and *N* as the number of mice per treatment group, with individual neuronal values averaged per mouse. Statistical analyses for patch‐clamp, ABR and immunolabelling data were performed using *N*, defined as the number of mice per treatment group. Interquartile range (IQR)‐based outlier detection was performed on each set of neurons, and identified outliers were removed prior to averaging the number of cells per animal. In the text, data are presented as the median and interquartile range (IQR: 25–75) or as the mean ± SD, depending on the data distribution. In the figures, data are presented as box plots showing the median with IQRs (box) and 10th–90th percentiles (whiskers) or as bar graphs showing means ± SD, depending on the data distribution. Statistical analyses of the data were performed with SigmaStat/SigmaPlot. Normality was tested by the Shapiro–Wilk test. Comparisons between different data sets were made depending on the distribution of the data using parametric tests for normally distributed data (Student's *t*‐test for comparing two groups and ANOVA with Holm–Sidak *post hoc* test for comparing three or more groups). When the normality assumption was violated, non‐parametric tests (Mann–Whitney *U*‐test for comparing two groups and Kruskal–Wallis ANOVA on ranks for comparing three or more groups) were used. Differences were considered statistically significant at *P* = 0.05 and presented in the figures as absolute *P*‐values. Intrinsic properties, in addition to postsynaptic current amplitudes and kinetics, were analysed using Stimfit software (Guzman et al., 2014). For immunohistochemical analyses, monaural noise exposure permitted internal paired comparisons (Student's paired *t*‐test). GlyT2 boutons and gephyrin puncta were quantified from ∼60 SPN neurons (30 per condition) across three mice, with values averaged per mouse (∼10 neurons per condition per mouse). Statistical significance for neural responses *in vivo* was assessed by using mixed linear models to account for between‐animal and within‐animal variability (python statsmodels MixedLM and SciPy for Normal CDF).

### Results

#### Induction of noise‐induced threshold shifts

Naïve C57BL/6J mice were subjected to a 2 h exposure of bandpass‐filtered noise (8–16 kHz) at 107 dB SPL, aiming at a moderate ∼40 dB threshold shift within the most sensitive range of the mouse audiogram (Bakay et al., 2018; Fernandez et al., 2015; Fischl et al., 2019; Ouagazzal et al., 2006; Zhang et al., 2013). Following noise exposure, animals were allocated to one of three experimental groups for subsequent experiments: immunohistochemistry; whole‐cell patch‐clamp recordings in brain slices; or *in vivo* single‐cell electrophysiology (Fig. 1*A*).

**Figure 1:**
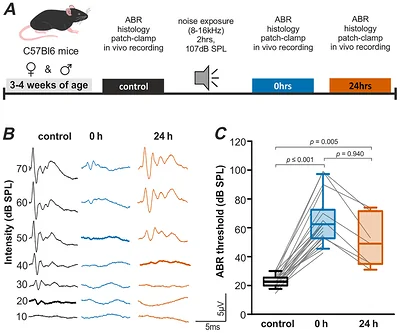
Auditory brainstem responses confirm noise‐induced threshold shifts *A*, experimental time line. *B*, representative ABR traces evoked by click stimuli before and after noise exposure. Bold lines indicate auditory thresholds. *C*, population average ABR thresholds show a significant threshold shift postexposure, confirming peripheral hearing loss. Grey lines depict threshold shifts of individual mice. *N* = 21 mice. Kruskal–Wallis ANOVA with Dunn's *post hoc* tests. Abbreviation: ABR, auditory brainstem response.

To confirm the efficacy of the noise protocol in inducing auditory threshold shifts, ABRs were recorded immediately before and directly after noise exposure. Representative traces of click‐evoked ABRs from pre‐exposure (control) and postexposure (0 h and 24 h) conditions are shown in Fig. 1*B*. This single 2 h noise exposure resulted in a significant elevation in ABR thresholds, indicative of substantial hearing loss. Average thresholds increased from 22.50 (IQR: 20–25) dB SPL at baseline to 62.50 (IQR: 52.75–72.50) dB SPL immediately postexposure (0 h), with little ABR recovery at 24 h postexposure (Kruskal–Wallis ANOVA with Dunn's *post hoc* tests, *N* = 21; Fig. 1*B* and 1*C*).

#### Noise exposure increases intrinsic excitability of SPN neurons

Auditory neurons, including those in the SPN, are typically quiescent in slice preparations in baseline conditions (Fig. 2*A*). Spontaneous action potential firing in the auditory brainstem is thought to originate primarily from the cochlea, particularly from Kolliker's organ during early development (Tritsch et al., 2007) and from inner hair cell‐driven synaptic activity without intrinsically evoked auditory nerve fibre firing in the mature system (Jaime Tobon & Moser, 2024; Wu, Young et al., 2016). In both cases, the preparation of acute brain slices for electrophysiological recordings inherently severs connections between the cochlea and central auditory structures, thereby eliminating peripheral sources of spontaneous activity. Consistent with this, most SPN neurons do not exhibit spontaneous firing at resting membrane voltages *in vitro* (Fig. 2*A*, left panel), with only some SPN neurons firing spontaneous action potentials at low frequencies in the brain slice (Fig. 2*A*, right panel). However, 24 h after noise exposure, there was a marked increase in spontaneous SPN activity, now with ∼30% of neurons (15/48) firing spontaneously and with active neurons showing significantly higher firing rates compared with control conditions (Kruskal–Wallis ANOVA with Dunn's *post hoc* tests; Fig. 2*A* and 2*C*). These findings suggest that noise exposure enhances intrinsic neuronal excitability in the SPN to enable spontaneous firing in the absence of afferent cochlear input.

**Figure 2:**
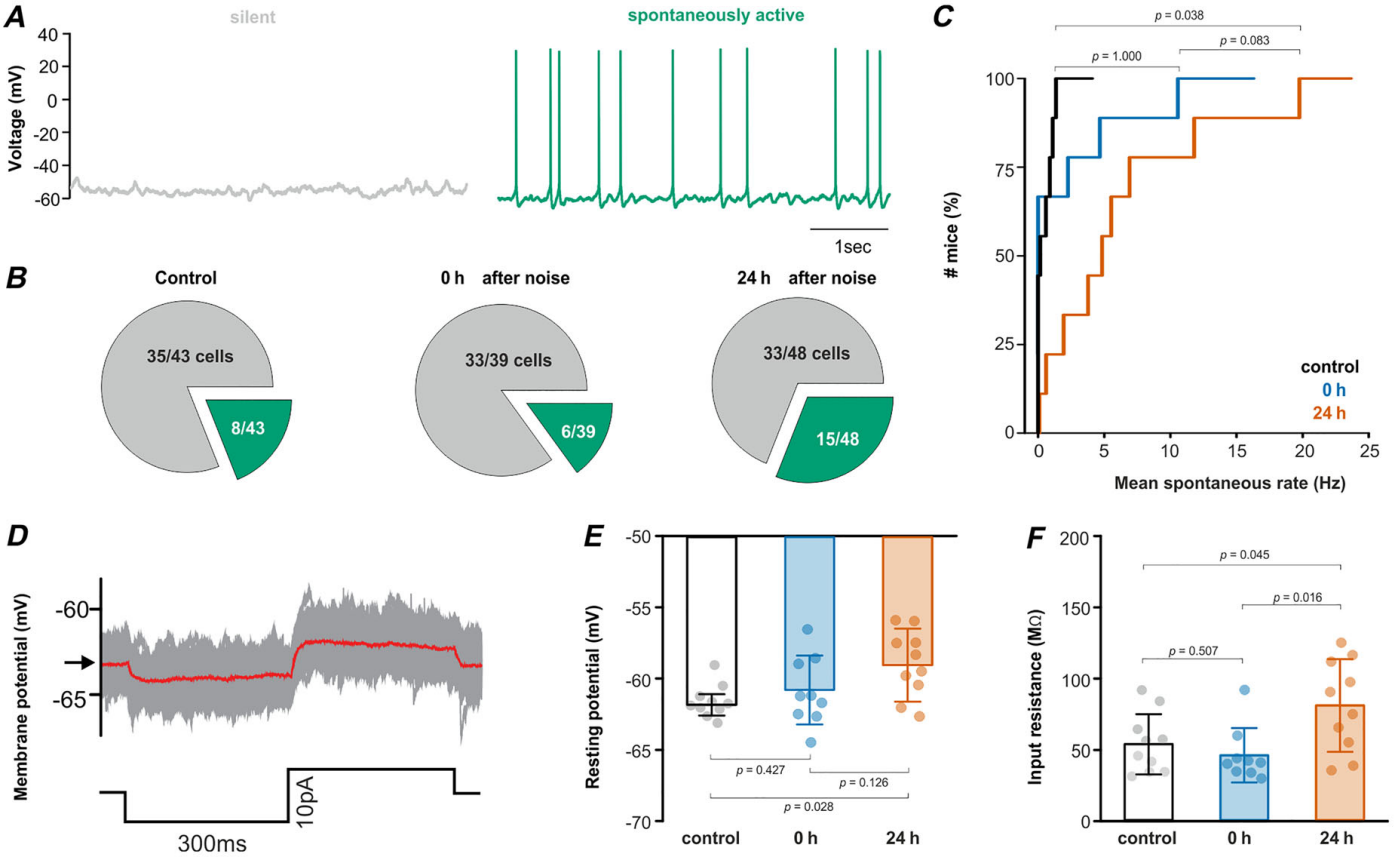
Noise exposure increases intrinsic excitability of superior paraolivary nucleus neurons *A*, representative voltage traces of SPN neurons exhibiting no spontaneous firing (silent) or spontaneous action potentials (spontaneously active). *B*, pie charts showing the changing proportions of silent *versus* active neurons following noise exposure. *C*, cumulative distribution of spontaneous firing frequencies in control conditions (black) and 0 h (blue) and 24 h (orange) post noise exposure, showing higher spontaneous firing rates 24 h postexposure (Kruskal–Wallis ANOVA with Dunn's *post hoc* tests). *D*, superimposed voltage responses to small biphasic current injections; average trace in red. Arrow indicates the resting membrane potential prior to current injection. *E*, population data for membrane potentials at control, 0 h and 24 h postexposure. Dots represent the number of mice. *F*, population data for input resistance (*R*_i_) calculated from steady‐state voltage responses shown in *D* using Ohm's law. Dots represent the number of mice. For data in *C*, *E* and *F*, control: *n* = 41 cells, *N* = 10 mice; 0 h: *n* = 37 cells, *N* = 9 mice; 24 h: *n* = 38 cells, *N* = 10 mice. ANOVA with Holm–Sidak *post hoc* tests. Abbreviation: SPN, superior paraolivary nucleus.

The intrinsic excitability of a neuron is governed by its passive membrane properties, including resting membrane potential (RMP) and input resistance (*R*_i_). In the present study, noise exposure significantly increased both RMP and *R*_i_, with higher *R*_i_ facilitating a greater voltage response to a given current input. The RMP of SPN neurons was recorded in the absence of current injection within 3–10 min after breaking into whole‐cell mode and was significantly more depolarized 24 h following noise exposure relative to both control and acute (0 h) conditions (control: −61.56 ± 1.14 mV, *n* = 42 cells, *N* = 10 mice; 0 h: −60.80 ± 2.42 mV, *n* = 39 cells, *N* = 9 mice; 24 h: −58.97 ± 2.40 mV, *n* = 48 cells, *N* = 10 mice; ANOVA with Holm–Sidak *post hoc* tests; Fig. 2*D* and 2*E*).

Input resistance was quantified by applying 50 repetitions of small‐amplitude (10 pA) current steps, and calculating *R*_i_ from the average steady‐state voltage deflection using Ohm's law (Fig. 2*D*). These minimal current injections were chosen to minimize activation of voltage‐gated conductances, thus isolating passive membrane properties. Noise exposure resulted in a significant increase in *R*_i_ at 24 h postexposure (control: 54.03 ± 21.10 MΩ, *n* = 41 cells, *N* = 10 mice; 0 h: 46.26 ± 19.10 MΩ, *n* = 37 cells, *N* = 9 mice; 24 h: 81.21 ± 32.46 MΩ, *n* = 38 cells, *N* = 10 mice; ANOVA with Holm–Sidak *post hoc* tests; Fig. 2*F*). Collectively, the depolarized RMP and the increased *R*_i_ suggest these intrinsic changes to drive the higher spontaneous firing rates observed following noise exposure.

#### Noise exposure increases the gain of offset and sustained firing rates of SPN neurons *in vitro*

Beyond passive membrane properties, neuronal excitability was assessed quantitatively by measuring both offset and sustained firing rates in response to intracellular current injection. In control and noise exposure groups, sustained depolarizing current injections between 50 and 950 pA elicited increasing action potential firing in SPN neurons. Injections of −500 pA hyperpolarized the membrane to −75 to −80 mV, a range readily produced by inhibitory inputs to SPN neurons and near the threshold for rebound spike generation at stimulus offset (Kopp‐Scheinpflug et al., 2011), a hallmark of SPN electrophysiology (Fig. 3*A*). Examination of rebound spiking near threshold provides an effective measure to study increased neural gain following noise exposure. This moderate hyperpolarization elicited near‐threshold rebound responses in 63% (26/41) of control cells, 61% (23/38) at 0 h, and 85% (41/48) at 24 h after noise exposure (Fig. 3*A*). At 24 h following noise exposure, SPN neurons exhibited a significant increase in both offset and sustained firing (Fig. 3*B*). Specifically, offset firing rates increased from 9.94 ± 5.56 Hz (*n* = 40 cells, *N* = 10 mice) in controls and 6.22 ± 4.01 Hz (*n* = 37 cells, *N* = 9 mice) at 0 h after noise exposure to 18.29 ± 8.35 Hz (*n* = 49 cells, *N* = 10 mice) at 24 h after exposure (ANOVA with Holm–Sidak *post hoc* tests; Fig. 3*C*). Likewise, sustained firing rates during depolarizing steps were elevated from 195.26 ± 89.80 Hz (*n* = 40 cells, *N* = 10 mice) in control neurons and 191.93 ± 101.19 Hz (*n* = 37 cells, *N* = 9 mice) at 0 h after noise exposure to 293.39 ± 62.47 Hz (*n* = 49 cells, *N* = 10 mice) at 24 h after exposure (ANOVA with Holm–Sidak *post hoc* tests; Fig. 3*D*). These data indicate a significant enhancement in neural gain of SPN neurons at 24 h after noise exposure.

**Figure 3:**
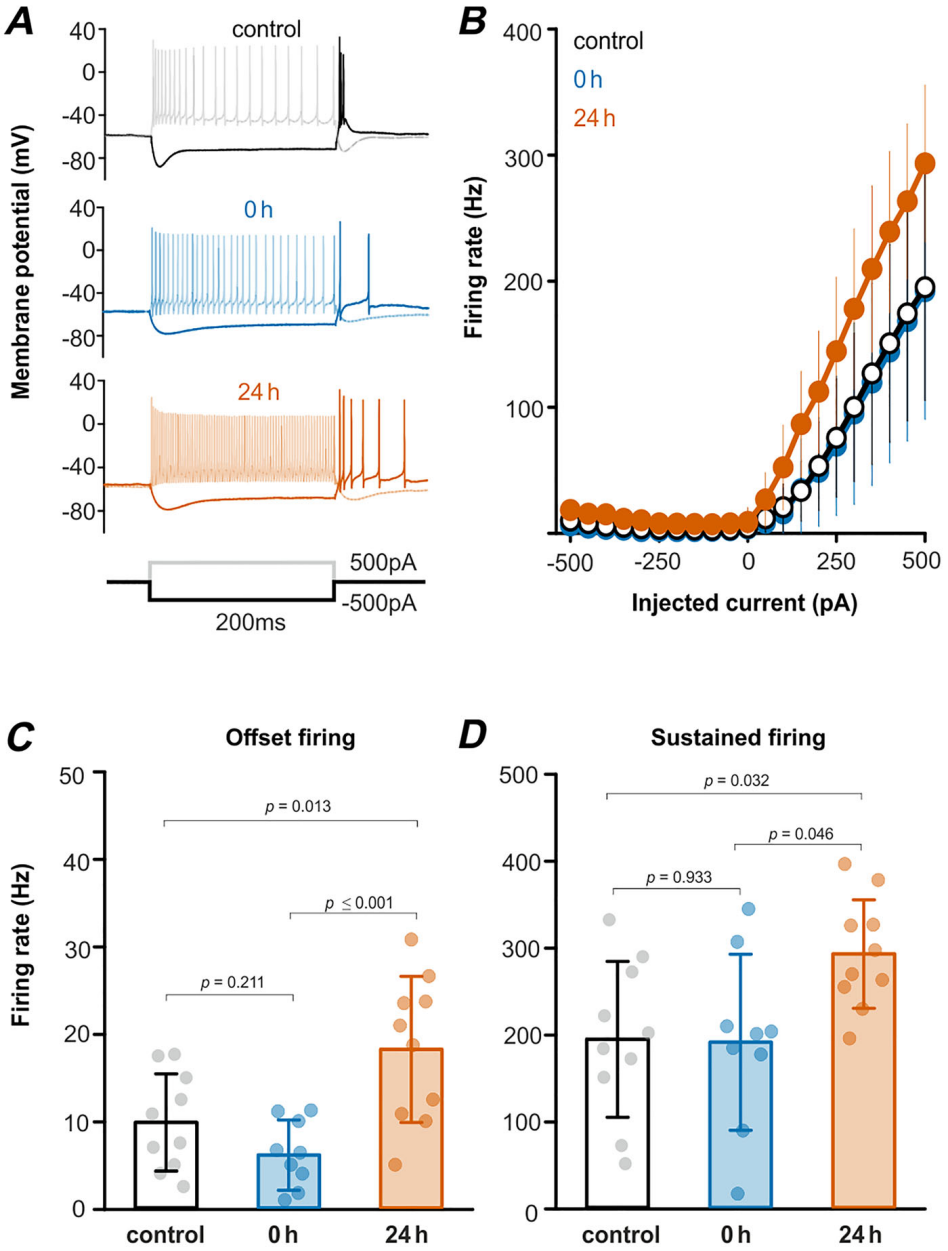
Noise exposure increases current‐evoked firing rates *A*, example voltage traces in response to −500 pA (opaque) and +500 pA (transparent) current injection for control (top trace), 0 h (middle trace) and 24 h (bottom trace), showed the characteristic rebound response at stimulus offset upon hyperpolarization and a sustained response upon depolarization. *B*, input–output functions show increased firing rates at 24 h following noise exposure. *C*, population data on offset firing in response to hyperpolarizing (−500 pA) current injections. *D*, population data on sustained firing in response to depolarizing (+500 pA) current injections. For data in *C* and *D*, dots represent the number of mice; control: *n* = 40 cells, *N* = 10 mice; 0 h: *n* = 37 cells, *N* = 9 mice; 24 h: *n* = 49 cells, *N* = 10 mice. ANOVA with Holm–Sidak *post hoc* tests.

Given that, at the neural circuit level, SPN OFF responses require a strong inhibitory input (Kopp‐Scheinpflug et al., 2011; Rajaram et al., 2019, 2020), we next tested for changes of synaptic inhibition in SPN neurons following noise exposure.

#### Increased spontaneous inhibitory synaptic transmission following noise exposure

Two mechanisms are frequently implicated in the enhanced central gain observed after noise‐induced hearing loss: increased intrinsic excitability and reduced synaptic inhibition (for a review, see Auerbach et al., 2014). Although we confirmed an increase in intrinsic excitability in SPN neurons following noise exposure, we next investigated how inhibitory synaptic transmission is affected in these neurons.

To assess changes in inhibitory input, we quantified mIPSCs in SPN neurons from control and noise‐exposed animals (Fig. 4*A*). Recordings were conducted at a holding potential of 0 mV to maximize the driving force for chloride‐mediated currents. Detected mIPSCs were identified by their asymmetric waveforms, with rapid rise and slower exponential decay, characteristic of synaptic currents (Fig. 4*A*, inset), and by their larger amplitudes in comparison to the electrical noise of the baseline (Fig. 4*B*). For each neuron, the frequency of mIPSCs was measured and found to be significantly increased at 24 h after exposure (control: 8.74 ± 4.15 Hz, *n* = 14 cells, *N* = 5 mice; 0 h: 12.42 ± 2.65 Hz, *n* = 22 cells, *N* = 9 mice; 24 h: 15.18 ± 5.28 Hz, *n* = 15 cells, *N* = 6 mice; ANOVA with Holm–Sidak *post hoc* tests; Fig. 4*C*).

**Figure 4:**
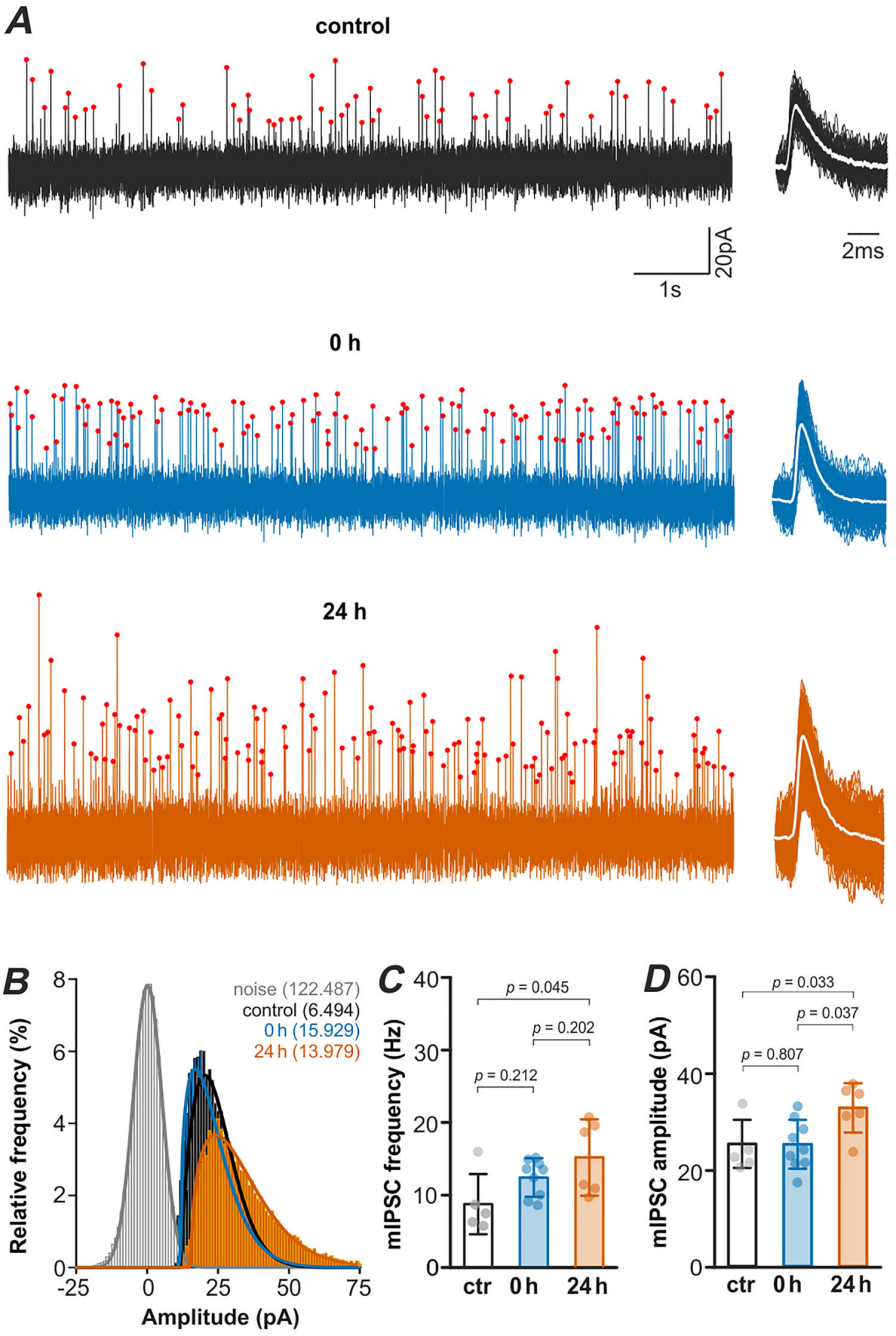
Noise exposure enhances the amplitude and frequency of miniature inhibitory postsynaptic currents in the superior paraolivary nucleus *A*, representative mIPSC traces from control (black), 0 h (blue) and 24 h (orange) postexposure neurons. Red dots indicate detected mIPSCs, shown superimposed to the right of the raw traces; white bold lines represent the average mIPSC waveform. *B*, frequency distributions of mIPSC peak amplitudes (1 pA bins) for control (black), 0 h (blue) and 24 h (orange) conditions. Bold lines indicate Gaussian fits; electrical noise is shown in grey. Event counts are given in parentheses. *C*, population data of mIPSC frequencies. *D*, population data of mIPSC amplitudes. For data in *C* and *D*, dots represent the number of mice; control: *n* = 14 cells, *N* = 5 mice; 0 h: *n* = 22 cells, *N* = 9 mice; 24 h: *n* = 15 cells, *N* = 6 mice. ANOVA with Holm–Sidak *post hoc* tests. Abbreviations: ctr, control; mIPSC, miniature inhibitory postsynaptic current; SPN, superior paraolivary nucleus.

We next analysed mIPSC amplitudes to assess potential postsynaptic changes in inhibitory synaptic transmission. Although there was no significant difference in amplitude immediately after exposure, a robust increase was observed at 24 h (control: 24.70 ± 5.32 pA, *n* = 14 cells, *N* = 5 mice; 0 h: 25.41 ± 5.05 pA, *n* = 22 cells, *N* = 9 mice; 24 h: 32.95 ± 5.10 pA, *n* = 15 cells, *N* = 6 mice; ANOVA with Holm–Sidak *post hoc* tests; Fig. 4*D*).

These findings demonstrate that, contrary to the frequently reported loss of inhibition following damaging noise exposure in other auditory nuclei, SPN neurons exhibit a significant increase in synaptic inhibition as early as 24 h postexposure.

#### Increased number of glycinergic boutons on SPN neurons following noise exposure

To assess structural changes in inhibitory synaptic input following noise exposure, high‐resolution confocal imaging was used to quantify synaptic terminals immunolabelled for the vesicular glycine transporter 2 (GlyT2), a presynaptic marker responsible for the re‐uptake of glycine from the synaptic cleft. Postsynaptic sites were identified by immunolabelling for gephyrin, a scaffolding protein associated with glycine receptor clustering (Fig. 5*A–C*).

**Figure 5:**
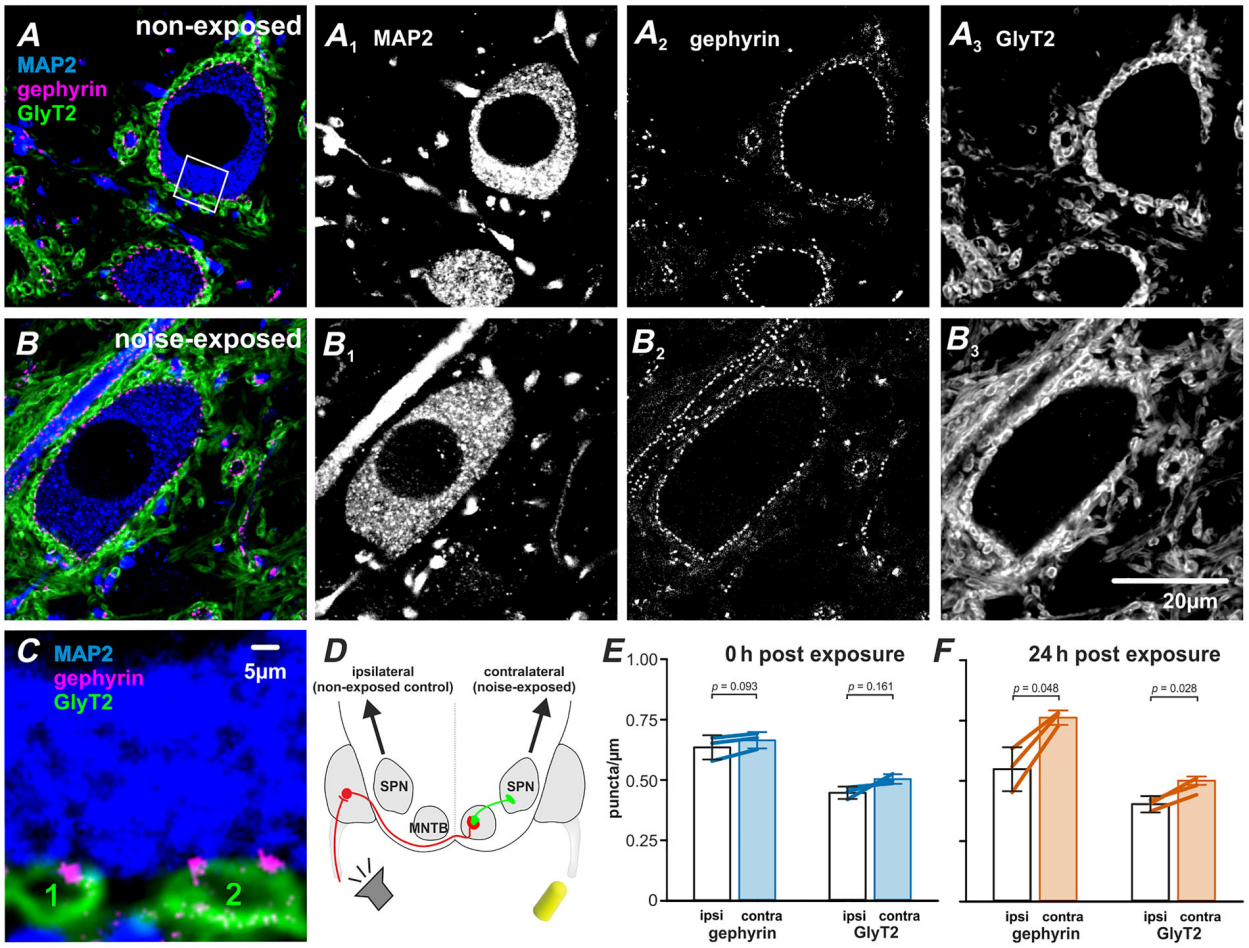
Glycine transporter type 2 and gephyrin expression in the superior paraolivary nucleus increases following noise exposure *A* and *B*, representative images showing MAP2 (blue), GlyT2 (green) and gephyrin (magenta) co‐labelling in control and noise‐exposed neurons. *C*, high‐magnification image of the neuronal membrane indicated in *A* used for quantification of gephyrin puncta and GlyT2 boutons. *D*, schematic diagram of unilateral noise exposure protocol for internal controls. *E*, gephyrin and GlyT2 densities normalized to postsynaptic membrane area immediately following noise exposure show no difference from control SPN. *F*, gephyrin and GlyT2 densities normalized to postsynaptic membrane area 24 h postexposure. For data in *E* and *F*, lines represent changes for individual animals; 0 h control side: *n* = 31 cells, *N* = 3 mice; 0 h exposed side: *n* = 30 cells, *N* = 3 mice; 24 h control side: *n* = 31 cells, *N* = 3 mice; 24 h exposed side: *n* = 30 cells, *N* = 3 mice. Student's paired *t*‐tests. Abbreviations: contra, contralateral; GlyT2, glycine transporter type 2; ipsi, ipsilateral; SPN, superior paraolivary nucleus.

The SPN neurons are selectively activated by auditory input from the contralateral ear. Therefore, by applying unilateral noise exposure while acoustically shielding the opposite ear, the SPN on the ipsilateral (non‐exposed) side serves as an internal control (Fig. 5*D*). This approach, albeit not perfect, facilitates more accurate and internally controlled quantification of immunohistochemical markers of synaptic inhibition, i.e. GlyT2‐ and gephyrin‐positive puncta (Fig. 5*E* and 5*F*).

To account for variability in neuronal soma size, the density of GlyT2‐ and gephyrin‐positive puncta was normalized to postsynaptic membrane length (in micrometres). At the acute time point (0 h), no significant difference was observed in the density of gephyrin‐positive puncta (control: 0.64 ± 0.05 puncta/µm, *n* = 31 cells, *N* = 3 mice; noise‐exposed: 0.67 ± 0.03 puncta/µm, *n* = 30 cells, *N* = 3 mice; Student's paired *t*‐test, *P* = 0.864) or GlyT2‐positive boutons (control: 0.45 ± 0.03 puncta/µm, *n* = 31 cells, *N* = 3 mice; noise‐exposed: 0.50 ± 0.03 puncta/µm, *n* = 30 cells, *N* = 3 mice; Student's paired *t*‐test; Fig. 5*E*). However, 24 h following noise exposure, both presynaptic and postsynaptic inhibition markers showed significant increases in density. Specifically, gephyrin‐positive puncta increased from 0.55 ± 0.09 puncta/µm on the control side (*n* = 31 cells, *N* = 3 mice) to 0.76 ± 0.03 puncta/µm on the noise‐exposed side (*n* = 30 cells, *N* = 3 mice; Student's paired *t*‐test), and GlyT2‐positive boutons increased from 0.40 ± 0.02 puncta/µm (*n* = 31 cells, *N* = 3 mice) to 0.49 ± 0.02 puncta/µm (*n* = 30 cells, *N* = 3 mice; Student's paired *t*‐test; Fig. 5*F*). To evaluate whether inhibitory markers could be reduced ipsilateral to noise exposure between 0 h and 24 h, we conducted an ANOVA with Holm–Sidak all‐pairwise multiple‐comparison *post hoc* testing across the four gephyrin groups and, independently, across the four GlyT2 groups. This analysis revealed no significant differences between the ipsilateral 0 h and 24 h conditions for either gephyrin (*P* = 0.187) or GlyT2 (*P* = 0.122), indicating no detectable time‐dependent reduction in inhibitory marker expression over this interval. Quantifying gephyrin and GlyT2 separately aimed at having two independent measures for anatomical changes in synaptic inhibition. Exclusively assessing sites with co‐localized GlyT2 and gephyrin could be an alternative approach to highlight functional synapses.

These data indicate that, in contrast to the often‐reported loss of inhibition following noise exposure, SPN neurons undergo a structural enhancement of glycinergic input, potentially aiding the generation of offset responses after noise exposure.

#### Noise exposure increases afferent fibre‐stimulated synaptic inhibition by enhanced bouton formation without altering the number of input fibres

To quantify physiologically how the number of inhibitory synaptic inputs to individual SPN neurons is altered following noise exposure, we measured IPSC amplitudes in response to graded electrical stimulation of medial nucleus of the trapezoid body (MNTB) axons. Gradual increases in IPSC amplitudes were observed with progressively stronger stimulation intensities (Fig. 6*A* and 6*B*), indicative of stepwise recruitment of individual presynaptic axons. To quantify the number of recruited axons objectively, we applied a density‐based clustering algorithm (DBSCAN), previously established to identify discrete amplitude steps corresponding to individual afferent input fibres in the SPN (Pedregosa et al., 2011; Rajaram et al., 2020). This analysis confirmed a convergence of approximately four MNTB axons onto each SPN neuron, consistent with existing literature (Lee et al., 2023; Rajaram et al., 2020). There was no statistically significant difference in the number of afferent input fibres between control animals (3.96 ± 0.99, *n* = 39 cells, *N* = 9 mice) and noise‐exposed animals at 24 h (4.12 ± 0.70, *n* = 33 cells, *N* = 12 mice; Student's paired *t*‐test; Fig. 6*C*). However, maximum IPSC amplitudes in SPN neurons increased significantly from 3.23 (IQR: 2.97–3.94) nA in controls (*n* = 39 cells, *N* = 9 mice) to 4.93 (IQR: 4.04–6.64) nA at 24 h after noise exposure (*n* = 33 cells, *N* = 12 mice, Mann–Whitney *U*‐test; Fig. 6*D*). Previous anatomical tracing studies showed that individual MNTB axons form multiple synaptic boutons on a single SPN target neuron (Rajaram et al., 2020). Our present data suggest that noise exposure leads to an expansion of the MNTB axonal arbor and increased synaptic bouton formation on SPN neurons, while the number of afferent MNTB neurons remains unchanged (Fig. 6*E*).

**Figure 6:**
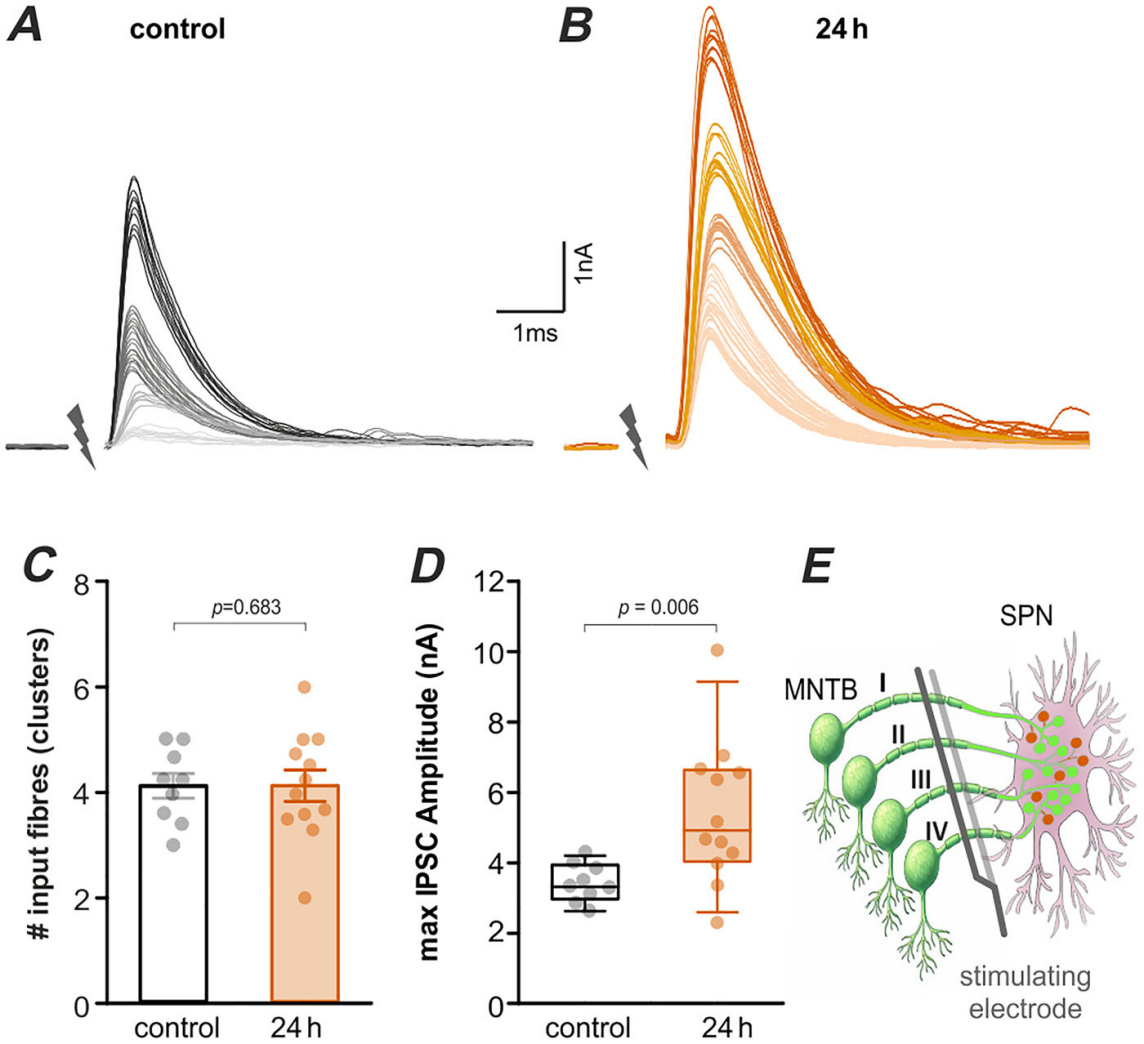
Noise exposure increases the inhibitory postsynaptic current amplitudes through increased numbers of synaptic boutons *A* and *B*, superimposed IPSCs showing a gradual increase in current amplitudes evoked by stepwise MNTB stimulation (10–100 V) for a control neuron (*A*) and a neuron at the 24 h postexposure time point. Electrical stimulus artefacts are removed for clarity. *C*, clustering analysis (DBSCAN) estimates approximately four MNTB inputs per SPN neuron, with unchanged by noise exposure. *D*, population data for maximum IPSC amplitudes. For data in *C* and *D*, dots in represent the number of mice; control: *n* = 39 cells, *N* = 9 mice; 24 h: *n* = 33 cells, *N* = 12 mice. Student's *t*‐tests. *E*, schematic diagram illustrating expanded MNTB axonal arbors and increased synaptic bouton formation on SPN neurons following noise exposure (depicted by additional orange boutons) compared with control (green boutons), without changes in the number of afferent fibres. Abbreviations: IPSC, inhibitory postsynaptic current; MNTB, medial nucleus of the trapezoid body; SPN, superior paraolivary nucleus.

#### Intrinsic and synaptic adaptations aid the recovery of SPN OFF responses 24 h after noise exposure

Our combination of *in vitro* electrophysiological and immunohistochemical approaches demonstrated that 24 h following noise exposure, SPN neurons exhibit increased intrinsic excitability alongside enhanced synaptic inhibition. To determine how these cellular changes act together to influence sound‐driven activity, we performed *in vivo* single‐unit recordings from SPN OFF‐responsive neurons in both control and noise‐exposed mice. SPN neurons were identified *in vivo* by: (1) their anatomical location between the lateral margin of the MNTB and the medial border of the lateral superior olive (LSO); (2) receiving strong contralateral inhibition and nearly no ipsilateral excitation; and (3) their characteristic action potential waveform (Fig. 7*D–F*), which is distinct from the MNTB owing to the lack of complex waveforms and from LSO neurons (Jing et al., 2021; Stancu et al., 2024).

**Figure 7:**
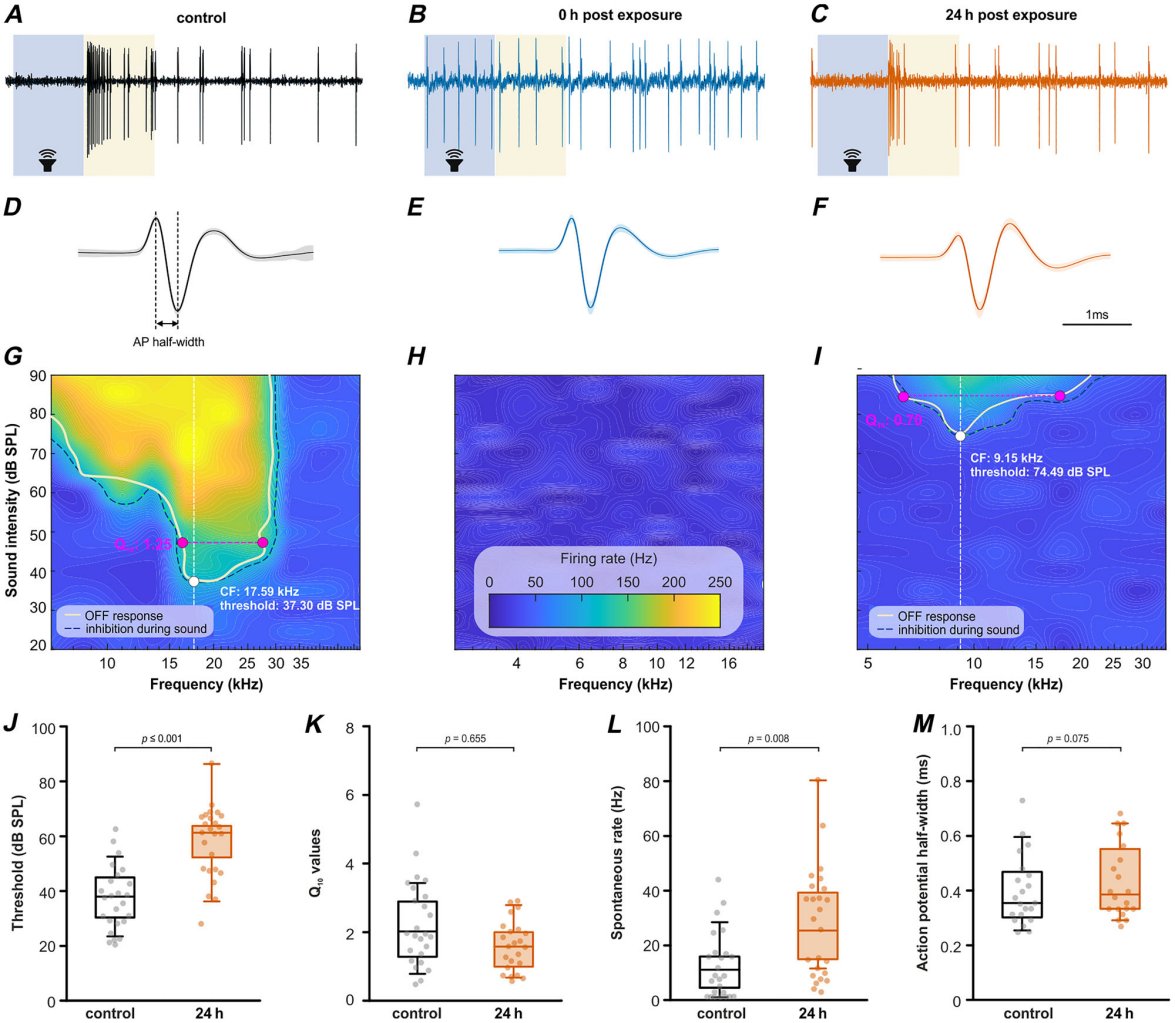
Loss and recovery of sound OFF responses in superior paraolivary nucleus neurons after noise exposure *A–C*, traces of extracellularly recorded action potentials during (blue shade) and after (yellow shade) sound presentations for control (*A*), 0 h (*B*) and 24 h (*C*) conditions. *D–F*, averaged waveforms of the cells shown in *A–C*. *G–I*, representative frequency–response maps from SPN neurons recorded at control (*G*), 0 h (*H*) and 24 h (*I*). Maps were obtained using pure tones centred on a cell‐specific reference frequency spanning ∼1.5 octaves above and below, with ≥10 logarithmically spaced frequency steps and 5 dB SPL intensity increments. Tuning curves (white continuous lines) were smoothed using modified Akima piecewise‐cubic interpolation (1000 × 1000 grid) and used to derive the CF, threshold and tuning sharpness (*Q*_10_ = CF/bandwidth at 10 dB above threshold). Blue dotted lines outline the response map derived from reduced activity during (rather than after) sound presentation. *J*, population auditory thresholds were significantly elevated at 24 h postexposure. *K*, population *Q*_10_ values showing no difference between control and noise‐exposed neurons. *L*, population spontaneous rates were significantly increased at 24 h postexposure. *M*, population data for action potential half‐width (as measured in *D*) showed no difference. For data in *J–M*, dots represent the number of cells; control: *n* = 25 cells, *N* = 12 mice; 24 h; *n* = 25 cells, *N* = 9 mice. Significance was tested by mixed linear models. Abbreviations: CF, characteristic frequency; *Q*_10_, tuning sharpness; SPN, superior paraolivary nucleus.

In control conditions, SPN neurons exhibited a moderate spontaneous firing rate, which was inhibited during sound presentation (Fig. 7*A*). Upon cessation of sound, neurons fired bursts of action potentials (OFF response) before the firing rate returned to spontaneous levels. At 0 h postexposure, no such OFF responses were encountered in the SPN. In a subset of experiments, SPN OFF neurons were recorded before noise exposure, followed by the 2 h noise exposure while maintaining the electrode position. Recordings then continued from the same SPN location that exhibited OFF responses prior to exposure. Despite ongoing spontaneous activity indicating viable neurons, OFF responses remained absent (Fig. 7*B*). Subsequent analysis was therefore restricted to control and 24 h time points. At 24 h postexposure, SPN OFF responses were observed again (Fig. 7*C*). The action potential shapes were similar across conditions, suggestive of recordings from the same cell types (Fig. 7*D–F*). Frequency–intensity response maps (Fig. 7*G–I*) depict significant increases in firing rates above spontaneous firing rates and were constructed from action potential firing during a 75 ms window following sound offset (yellow shade in Fig. 7*A–C*). The resulting tuning curves (white outline in Fig. 7*G* and *I*) were used to assess the CF, threshold and tuning sharpness (*Q*_10_) of neurons. Composing the frequency–intensity response maps for the 100 ms time window during sound presentation (blue shade in Fig. 7*A–C*) yielded a similar, yet inverted map outline owing to the inhibition of action potential firing during sound. The outlines of these inhibitory tuning curves are shown for comparison (blue dashed line in Fig. 7*G* and 7*I*). At 0 h postexposure, none of the neurons showed a significant increase in firing rates (Fig. 7*B* and 7*H*). Although peripheral thresholds had not recovered fully by 24 h, OFF responses were again observed in the SPN (Fig. 7*C* and 7*I*). Noise exposure led to a significant elevation in OFF response thresholds, increasing from 37.69 (IQR: 28.49–44.73) dB SPL in control neurons (*n* = 25 cells, *N* = 12 mice) to 61.26 (IQR: 47.53– 67.50) dB SPL in noise‐exposed animals at 24 h (*n* = 25 cells, *N* = 9 mice, mixed linear model statistics; Fig. 7*J*). Despite this shift in threshold, tuning sharpness, as measured by *Q*_10_ values, was not significantly affected [control: 1.93 (IQR: 1.26–3.16); 24 h: 1.60 (1.02–2.09); linear mixed model statistics; Fig. 7*K*], suggesting that frequency tuning remained intact but shifted to higher intensities, rather than cutting off the tip of the tuning curve. Following noise exposure, a significant increase in spontaneous firing rates was observed in exposed mice [24 h: 29.68 (IQR: 9.22–41.42) Hz; *n* = 25 cells, *N* = 9 mice] compared with control animals [control: 8.96 (IQR: 1.00–16.85) Hz; *n* = 25 cells, *N* = 12 mice, mixed linear model statistics; Fig. 7*L*], consistent with enhanced excitability previously demonstrated in acute brain slice recordings (Fig. 2). The time between the peak and trough of the extracellular waveform is an established measure for action potential half‐width (Ritzau‐Jost et al., 2021). Noise exposure did not induce significant changes in the half‐width of extracellularly recorded SPN action potentials [control: 0.35 (IQR: 0.30–0.47) ms, *n* = 21 cells, *N* = 11 mice; 24 h: 0.39 (IQR: 0.33–0.55) ms, *n* = 20 cells, *N* = 9 mice; mixed linear model statistics; Fig. 7*M*].

#### Recovery of OFF response temporal dynamics 24 h following noise exposure

The intensity‐dependent temporal characteristics of OFF responses were altered following noise exposure. Dot raster plots of control SPN neurons show the characteristic increase in action potential firing at sound offset that strengthens with increasing sound intensity (Fig. 8*A*). At 24 h following noise exposure, 14/25 neurons displayed robust OFF responses at high intensities (Fig. 8*B*). In contrast, 11/25 neurons were strongly inhibited during sound presentation but lacked a distinct OFF response, showing no increase in firing at sound offset (Fig. 8*C*). These inhibition‐only neurons exhibited highly variable latencies that did not shorten systematically with increasing sound level, consistent with a mere resumption of spontaneous firing after peristimulus inhibition (Fig. 8*D*). Apparent differences in latency between control and 24 h postexposure OFF‐responsive neurons were eliminated after correcting for threshold, revealing identical latencies at matched sensation levels (Fig. 8*E*).

**Figure 8:**
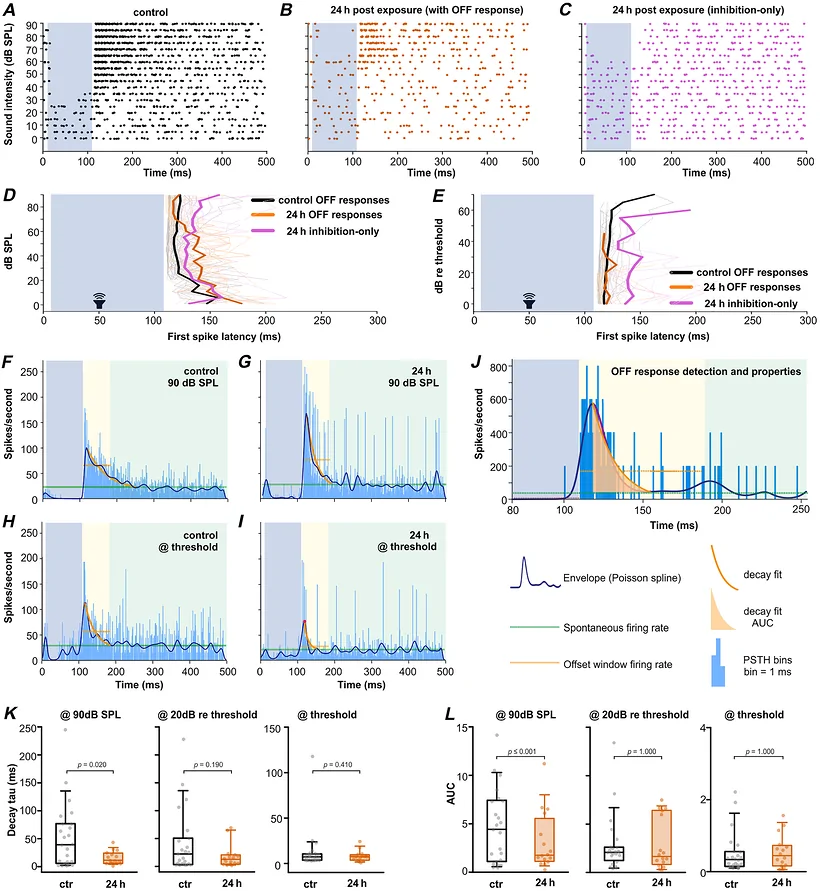
Changes in superior paraolivary nucleus OFF response dynamics following noise exposure *A* and *B*, example raster plots from typical neurons in control conditions (*A*) and 24 h postexposure (*B*) in response to five repetitions of 100 ms tone bursts at the CF, with intensities of ≤90 dB SPL. Blue shading indicates the stimulus window. Spikes within 75 ms after stimulus offset were used to quantify OFF responses. *C*, example raster of inhibition‐only neurons, showing peristimulus inhibition and absence of an OFF response. *D*, mean OFF response latency as a function of stimulus intensity for control (black), noise‐exposed with OFF response (orange) and inhibition‐only (purple) neurons. *E*, same design as *D*. OFF response latency was corrected for stimulus intensity relative to threshold. *F* and *G*, population PSTHs (1 ms bins) at CF and 90 dB SPL for control neurons (*F*) and noise‐exposed neurons (*G*), showing robust OFF responses. *H* and *I*, same design as *F* and *G*, but at CF and 20 dB above threshold. *J*, firing rates (in spikes per second) were computed by dividing spike counts by the bin width (1 ms) and number of repetitions. Continuous orange lines indicate exponential decay fits (τ); orange shaded areas denote the AUC. *K* and *L*, population data for PSTH decay time constants (*K*) and OFF‐response AUCs (*L*). For data in *D–K*, dots represent the number of cells; control: *n* = 24 cells, *N* = 11 mice; 24 h: *n* = 14 cells, *N* = 8 mice. Significance was tested by mixed linear models. Abbreviations: AUC, area under the curve; CF, characteristic frequency; ctr, control; PSTH, post‐stimulus time histogram; SPN, superior paraolivary nucleus.

Analysis of OFF response dynamics was restricted to control neurons and those that did exhibit OFF responses at the 24 h time point (Fig. 8*B*). Grand average PSTHs in response to 90 dB SPL tones revealed robust OFF responses in both groups (Fig. 8*F* and 8*G*). However, the temporal profile (Fig. 8*J*) of these OFF responses was significantly altered for responses to high sound intensities (Fig. 8*F* and *G*). Specifically, the decay time constants of the OFF responses were markedly shorter in noise‐exposed animals [11.55 (IQR: 5.74–24.20) ms, *n* = 14 cells, *N* = 8 mice] relative to controls [39.27 (IQR: 5.95–76.85) ms, *n* = 24 cells, *N* = 11 mice; mixed linear model statistics; Fig. 7*F*, 7*G* and 7*K*]. This accelerated decay, in conjunction with elevated spontaneous firing, resulted in an overall reduction in OFF response magnitude, as quantified by the area under the curve (AUC) of the PSTH [control: 4.65 (1.13–7.39), *n* = 24 cells, *N* = 11 mice; 24 h: 1.76 (1.13–5.56), *n* = 14 cells, *N* = 8 mice; mixed linear model statistics; Fig. 8*F*, 8*G* and 8*L*].

Given that shifts in auditory threshold induced by noise exposure are likely to arise upstream of the SPN, we assessed OFF response dynamics at equal sensation levels (20 dB above threshold and at threshold) to control for peripheral hearing loss. PSTHs obtained in these adjusted stimulus conditions (control: Fig. 8*H*; noise‐exposed: Fig. 8*I*) revealed more comparable response profiles between groups. At 20 dB above threshold, the decay time constants [control: 22.17 (3.33–33.87) ms, *n* = 24 cells, *N* = 11 mice; 24 h: 12.77 (IQR: 3.59–22.63) ms, *n* = 14 cells, *N* = 8 mice; linear mixed model statistics, Fig. 8*L*] and AUC values [control: 2.07 (IQR: 1.08–2.61); 24 h: 1.66 (IQR: 0.76–6.11); mixed linear models, *P* = 1; Fig. 8*K*] no longer differed significantly between groups.

Collectively, these findings suggest that although noise exposure induces marked changes in spontaneous activity and OFF response kinetics at absolute sound levels, the fundamental properties of suprathreshold OFF responses, including their spectral and temporal characteristics, are recovered when accounting for peripheral threshold shifts. This observation might reflect a compensatory balance between increased intrinsic excitability and enhanced synaptic inhibition within the SPN circuit to secure sound OFF encoding.

### Discussion

The aim of the present study was to determine how noise exposure effects neurons that are driven solely by inhibition. Sound OFF responses in the SPN are generated *de novo* via a highly effective inhibitory rebound mechanism (Kopp‐Scheinpflug et al., 2011, 2018). *In vivo*, SPN OFF responses were abolished at 0 h after noise exposure. Within the next 24 h, SPN neurons adapted by an increase in input resistance and a depolarization of the RMP. Together, these changes contribute to an increase in spontaneous and current‐evoked neuronal activity in acute brain slice preparations. In addition, noise exposure significantly enhances inhibitory synaptic transmission. Increased intrinsic excitability combined with enhanced inhibition supports the recovery of SPN OFF responses by 24 h after noise exposure. We used a single 2 h exposure to 8–16 kHz bandpass noise at 107 dB SPL to induce a moderate threshold shift (up to ∼40 dB) within the most sensitive region of the mouse audiogram (Bakay et al., 2018; Fernandez et al., 2015; Fischl et al., 2019; Ouagazzal et al., 2006; Zhang et al., 2013). Similar protocols have been shown to cause inner hair cell synaptopathy, despite recovery of ABR thresholds after ∼4 weeks (Bakay et al., 2018; Fernandez et al., 2015). In the present study, we focused on short‐term adaptations within 24 h, because these early changes might reveal targets for immediate intervention following damaging noise exposure.

#### Alterations in neural gain and intrinsic excitability

Neuronal *R*_i_ at rest is a conventional indicator of neuronal gain and excitability, because it reflects the number of ion channels open at the RMP (Mishra & Narayanan, 2025). The primary ion channels contributing to RMP include potassium leak channels, low‐voltage‐activated potassium channels and hyperpolarization‐activated cyclic nucleotide‐gated (HCN) channels, the last of which are highly expressed in SPN neurons (Felix et al., 2011; Kopp‐Scheinpflug et al., 2011, 2015). Noise exposure has been shown to activate neuromodulators, such as nitric oxide, in the auditory brainstem (Coomber et al., 2014, 2015; Hockley et al., 2020), which, in turn, reduces HCN channel activity in the SPN (Kopp‐Scheinpflug et al., 2015). The inward current through HCN channels depolarizes the membrane potential, thereby bringing the neuron closer to the firing threshold and increasing excitability. Paradoxically, HCN channel opening also increases membrane conductance, which lowers *R*_i_ and reduces excitability; an antagonism that has been reviewed recently (Mishra & Narayanan, 2025). As articulated by Mishra & Narayanan (2025): ‘The conductance and the current mediated by resting HCN channels, therefore, act in opposite directions in terms of how they regulate excitability’. Therefore, the net effect of HCN channel activity on excitability is likely to depend on the cell type and the physiological context. Possible contributions to RMP in SPN neurons by other conductances and their adaptation to noise exposure need to be addressed in future studies.

#### Synaptogenesis and increased inhibitory drive

Noise exposure can enhance synaptic strength and increase miniature postsynaptic current frequency at various auditory brainstem synapses, including the ventral cochlear nucleus and the medial superior olive (MSO) (Siveke et al., 2021; Wong & Xu‐Friedman, 2022). These changes might result from alterations in release probability, expansion of the readily releasable vesicle pool or the formation of new synapses. In monaural ventral cochlear nucleus bushy cells, for example, noise exposure leads to increased strength of endbulb of Held excitatory synapses, which was attributed to an increased number of synaptic contacts (Wong & Xu‐Friedman, 2022).

Other reports, such as those involving neurons in the LSO, have found decreases in both excitatory and inhibitory inputs following noise exposure (Pilati et al., 2016). Despite such variability, the overall balance between excitation and inhibition in binaural neurons appears to be maintained across different auditory brainstem nuclei, regardless of whether absolute input levels increase or decrease (Pilati et al., 2016; Siveke et al., 2021). Interestingly, neurons in the SPN, LSO and MSO all receive inhibitory input from the MNTB, yet following noise exposure, mIPSCs increase in the SPN and MSO, but decrease in the LSO (Pilati et al., 2016; Siveke et al., 2021). This strengthens the notion of circuit‐specific adaptations of inhibitory input to best match the functional necessities of the computations performed by each nucleus.

In the present study, we show that SPN neurons, whose action potential output is driven predominantly by inhibitory input, respond to noise exposure with an increase in inhibitory synaptic strength. This enhancement was likewise associated with an increase in the number of inhibitory synapses. An intriguing question is how new inhibitory synapses are formed in the mature SPN, given that the developmental establishment of inhibitory circuits typically requires a depolarizing chloride gradient that enables activity‐dependent ‘fire and wire’ mechanisms (Katz & Shatz, 1996; Rivera et al., 2005). Notably, in the SPN, both the chloride gradient and inhibitory synaptic architecture are established early in postnatal development (Blaesse et al., 2005; Lee et al., 2023; Lohrke et al., 2005; Rajaram et al., 2020). Future studies might explore whether spontaneous firing or rebound spiking, triggered by single spontaneous inputs of hyperpolarizing inhibition, is sufficiently robust and temporally precise to support activity‐dependent synaptogenesis in mature SPN neurons (Matsumoto et al., 2024). This idea is supported by recent evidence showing that even a single IPSC can evoke rebound firing in SPN neurons (Franken et al., 2025).

#### Downstream OFF responses

Sound‐offset (OFF) responses have been documented extensively in auditory centres downstream of the SPN, including the inferior colliculus (Akimov et al., 2017; Heeringa & van Dijk, 2014; Kasai et al., 2012; Parker et al., 2025; Wong & Borst, 2019), the medial geniculate body (Anderson & Linden, 2016; He, 2001, 2002; Yu et al., 2004) and the auditory cortex (Baba et al., 2016; Liu et al., 2019; Scholl et al., 2010; Solyga & Barkat, 2019). Notably, these downstream OFF responses often exhibit both spectral and spatial asymmetries relative to the responses of neurons during sound presentation (i.e. ON responses). Specifically, the frequency tuning of OFF responses is typically shifted towards higher frequencies in comparison to the CF of the neuron, although in some instances it is shifted lower (Anderson & Linden, 2016; He, 2002; Scholl et al., 2010).

This spectral asymmetry can be explained by a model in which neurons receive sharply tuned excitatory inputs combined with more broadly tuned inhibitory inputs, resulting in the formation of inhibitory sidebands. Within these sidebands, the excitatory–inhibitory balance is biased towards inhibition, leading to suppression of firing during sound presentation at these OFF‐CFs. Following sound cessation, and assuming that appropriate intrinsic membrane conductances are present, neurons may exhibit post‐inhibitory rebound spiking preferentially for sideband frequencies, giving rise to asymmetric OFF responses. A similar mechanism might underlie spatial asymmetries, potentially emerging from broad inhibitory projections across isofrequency laminae in the inferior colliculus, which modulate activity over a wider spatial domain (Parker et al., 2025).

In contrast, OFF responses in the SPN display spectral symmetry with ON responses: the same frequency that elicits a reduction in firing during sound presentation (ON inhibition) evokes an increase in firing upon sound termination (OFF excitation). This mirrored response profile effectively constitutes a ‘negative image’ of the ON response. Such a representation might confer functional advantages for auditory processing. By generating an internal prediction or inverse representation of expected sensory input, the auditory system can enhance contrast between expected and deviant stimuli. This contrast enhancement might facilitate more accurate stimulus detection and classification by suppressing redundant or predictable components of the input, thereby emphasizing novel or behaviourally relevant features.

#### Suprathreshold OFF responses following noise exposure

Noise exposure was shown preferentially to affect auditory nerve fibres with low spontaneous rates and high thresholds. However, recent studies in mice suggest that different auditory nerve fibre subtypes might be impacted more uniformly than previously thought, indicating that high‐threshold fibres might have equal survival rates to low‐threshold fibres (Furman et al., 2013; Suthakar & Liberman, 2021). This more balanced vulnerability and survival could contribute to the recovery of suprathreshold OFF responses in SPN neurons following noise exposure.

Our data demonstrate that at an absolute sound level of 90 dB SPL, the magnitude of OFF responses in the SPN is significantly reduced 24 h following noise exposure. Given that the SPN projects to the inferior colliculus (Gonzalez‐Hernandez et al., 1996; Nordeen et al., 1983; Saldana & Berrebi, 2000), it is likely that these attenuated OFF responses are also reflected in inferior colliculus activity. Indeed, a recent study using a comparable noise exposure protocol reported such a reduction in inferior colliculus OFF responses at the 24 h postexposure time point (Parker et al., 2025). To gain a better understanding of the underlying mechanisms of these changes, we analysed neural activity relative to the threshold for each neuron rather than using fixed absolute sound levels. This approach offers a more mechanistic perspective of synaptic transmission and postsynaptic integration, because it accounts for individual variations in excitability and hearing thresholds. When SPN responses were assessed at 20 dB above threshold, we observed no significant difference in OFF response dynamics between noise‐exposed and control animals, suggesting a partial functional recovery within 24 h. This recovery is likely to be mediated by adaptive mechanisms, including enhanced inhibitory synaptic strength and increased intrinsic excitability in SPN neurons.

These findings indicate that although absolute responses to high‐intensity stimuli are initially abolished following noise exposure, adaptive compensatory mechanisms might enable a rapid re‐establishment of functional OFF responses in the auditory brainstem. Future studies are needed to track the temporal progression of this recovery and to determine how these changes propagate along the ascending auditory pathway. Our observation of postexposure SPN responses showing strong peristimulus inhibition but without a subsequent OFF response might suggest incomplete recovery of OFF responses in these neurons. In these inhibition‐only neurons, the enhancement of inhibition might precede the increase in intrinsic excitability.

Recovery or preservation mechanisms for OFF responses might also play a role in the ageing auditory system, because robust OFF responses have been observed in aged non‐human primates. There, age‐related changes in the auditory cortex predominantly impaired ON responses, while OFF responses were relatively well preserved (Ramamurthy & Recanzone, 2020). OFF responses are believed to play a critical role in the temporal resolution needed in the detection of silent gaps in sound, which is a key feature of speech processing. OFF responses are also essential for encoding rapid intensity transients necessary to understand speech in complex acoustic environments, making their stabilization in the face of acoustic trauma or age‐related hearing loss crucially important (Awwad et al., 2023; Weible et al., 2014). Interestingly, auditory enrichment can prevent noise‐induced deficits in gap detection and, possibly, understanding speech in noise (Sturm et al., 2017). Given that OFF responses are crucial for gap detection, it remains to be determined whether auditory enrichment, as demonstrated by Sturm and colleagues, can likewise promote the restoration of OFF responses after noise exposure or during ageing. Such enrichment protocols might enhance network excitability, inhibitory balance or temporal precision within neural circuits, thereby supporting recovery of sound‐offset signalling. Investigating whether targeted auditory experience can preserve or reinstate OFF responses could provide important insight into activity‐dependent plasticity mechanisms and inform non‐invasive intervention strategies for possible noise‐ or age‐related OFF‐response processing deficits (Lu & Recanzone, 2026; Parker et al., 2025).

## Additional information

## Competing interests

The authors declare no competing financial interests.

## Author contributions

All experiments were performed at the Biocenter of the Ludwig‐Maximilians‐University Munich, Germany. B.G. and C.K‐S. designed the research. M.S, E.R., J.A.K. and C.K‐S. acquired, analysed and interpreted the data. M.S., E.R., J.A.K., B.G. and C.K‐S. contributed to writing the manuscript. All authors approved the final version of the manuscript and agree to be accountable for all aspects of the work in ensuring that questions related to the accuracy or integrity of any part of the work are appropriately investigated and resolved. All persons designated as authors qualify for authorship, and all those who qualify for authorship are listed.

## Funding

This work was funded by the DFG (KO2207/3‐1), the Munich Cluster for Systems Neurology (EXC 2145: Project‐ID 39 085) and the Graduate School of Systemic Neurosciences (GSN: GS‐82).

## Supporting information


Peer Review History

## Data Availability

Data are available from the corresponding author upon request.
