# Supplementary material for: Noise‐induced reduction and early recovery of superior paraolivary nucleus sound‐offset responses
Source: J Physiol. 2026 Apr 16;604(21):8796–816. doi: 10.1113/JP289987 (PMC13725237; doi:10.1113/JP289987)
Supplement: Supplementary file 1 [file TJP-604-8796-s001.pdf]

The Journal of Physiology

Noise-Induced Reduction and Early Recovery of SPN OFF Responses

Mihai Stancu, Ezhilarasan Rajaram, Joseph A. Kroeger, Benedikt Grothe, and Conny Kopp-Scheinpflug
DOI: 10.1113/JP289987

Corresponding author(s): Conny Kopp-Scheinpflug (cks@bio.lmu.de)

The referees have opted to remain anonymous.

Review Timeline:

Submission Date:	10-Sep-2025
Editorial Decision:	07-Oct-2025
Revision Received:	01-Feb-2026
Editorial Decision:	12-Mar-2026
Revision Received:	23-Mar-2026
Editorial Decision:	25-Mar-2026
Revision Received:	26-Mar-2026
Accepted:	01-Apr-2026

Senior Editor: Vaughan Macefield

Reviewing Editor: Karin Dedek

Transaction Report:

(Note: With the exception of the correction of typographical or spelling errors that could be a source of ambiguity, letters and reports are not edited. Depending on transfer agreements, referee reports obtained elsewhere may or may not be included in this compilation. Referee reports are anonymous unless the Referee chooses to sign their reports.)

Re: JP-RP-2025-289987 "**Noise-Induced Reduction and Early Recovery of SPN OFF Responses**" by Mihai Stancu, Ezhilarasan Rajaram, Joseph Kroeger, Benedikt Grothe, and Conny Kopp-Scheinpflug

Dear Conny,

Thank you for submitting your manuscript to The Journal of Physiology. It has been assessed by a Reviewing Editor and by 2 expert referees and we are pleased to tell you that it is potentially acceptable for publication following satisfactory major revision.

Please advise your co-authors of this decision as soon as possible.

The referee reports are copied at the end of this email.

Please address all the points raised and incorporate all requested revisions or explain in your Response to Referees why a change has not been made. We hope you will find the comments helpful and that you will be able to return your revised manuscript within 2 months. If you require an extension, please contact journal staff: jp@physoc.org. Please note that this letter does not constitute a guarantee for acceptance of your revised manuscript.

Your revised manuscript should be submitted online using the link in your Author Tasks: Link Not Available. This link is accessible via your account as Corresponding Author; it is not available to your co-authors. If this presents a problem, please contact journal staff (jp@physoc.org). Image files from the previous version are retained on the system. Please ensure you replace or remove any files that are being revised.

If you do not wish to submit a revised version of your manuscript, you must inform our journal staff (jp@physoc.org) or reply to this email to request withdrawal. Please note that a manuscript must be formally withdrawn from the peer review process at one journal before it may be submitted to another journal.

TRANSPARENT PEER REVIEW POLICY: To improve the transparency of its peer review process, The Journal of Physiology publishes online as supporting information the peer review history of all articles accepted for publication. Readers will have access to decision letters, including Editors' comments and referee reports, for each version of the manuscript, as well as any author responses to peer review comments. Referees can decide whether or not they wish to be named on the peer review history document.

ABSTRACT FIGURES: Authors are expected to use The Journal's premium BioRender account to create/redraw their Abstract Figures. Information on how to access this account is here: https://physoc.onlinelibrary.wiley.com/journal/14697793/biorender-access.

This will enable Authors to create and download high-resolution figures. If authors have used the free BioRender service, they can use the instructions provided in the link above to download a high-resolution version suitable for publication.

The link provided should only be used for the purposes of this submission. Authors will be charged for figures created on this account if they are not related to this manuscript submission.

LANGUAGE EDITING AND SUPPORT FOR PUBLICATION: If you would like help with English language editing, or other article preparation support, Wiley Editing Services offers expert help, including English Language Editing, as well as translation, manuscript formatting, and figure formatting at www.wileyauthors.com/eeo/preparation. You can also find resources for Preparing Your Article for general guidance about writing and preparing your manuscript at www.wileyauthors.com/eeo/prepresources.

RIGOUR AND REPRODUCIBILITY: We recommend authors consult The Journal's Rigour and Reproducibility requirements to ensure that their manuscript is suitable for publication: https://physoc.onlinelibrary.wiley.com/pb-assets/hub-assets/physoc/documents/TJP-Rigour-and-Reproducibility-Requirements.pdf

REVISION CHECKLIST:

Check that your Methods section conforms to journal policy: https://jp.msubmit.net/cgi-bin/main.plex?form_type=display_requirements#methods.

Check that data presented conforms to the statistics policy: https://jp.msubmit.net/cgi-bin/main.plex?form_type=display_requirements#statistics.

Upload a full Response to Referees file. To create your 'Response to Referees': copy all the reports, including any comments from the Senior and Reviewing Editors, into a Microsoft Word, or similar, file and respond to each point, using font or background colour to distinguish comments and responses and upload as the required file type.

Please upload two versions of your manuscript text: one with all relevant changes highlighted and one clean version with no

changes tracked. The manuscript file should include all tables and figure legends, but each figure/graph should be uploaded as separate, high-resolution files.

You may also upload:

- 'Potential Cover Art' for consideration as the issue's cover image
- Appropriate Supporting Information (Video, audio or data set: see https://jp.msubmit.net/cgi-bin/main.plex?form_type=display_requirements#supp).

We look forward to receiving your revised submission.

If you have any queries, please reply to this email and we will be pleased to advise.

Best wishes,

Vaughan Macefield
Senior Editor
The Journal of Physiology

REQUIRED ITEMS

- Author photo and profile. First or joint first authors are asked to provide a short biography (no more than 100 words for one author or 150 words in total for joint first authors) and a portrait photograph. These should be uploaded and clearly labelled together in a Word document with the revised version of the manuscript. See Information for Authors for further details.

- You must start the Methods section with a paragraph headed Ethical approval (https://jp.msubmit.net/cgi-bin/main.plex?form_type=display_requirements#methods).

Research must comply with The Journal's policies regarding animal experiments (https://physoc.onlinelibrary.wiley.com/hub/animal-experiments) and adherence to these policies must be stated in the manuscript.

Authors should confirm in their Methods section that their experiments were carried out according to the guidelines laid down by their institution's animal welfare committee, including an ethics approval reference number. The Methods section must contain a statement about access to food, water and housing, details of the anaesthetic regime: anaesthetic used, dose and route of administration, and method of killing the experimental animals.

- Your manuscript must include a complete Additional Information section, including competing interests; funding; author contributions and acknowledgements.

- Please upload separate high-quality figure files via the submission form.

- Please ensure that the Article File you upload is a Word file.

- Papers must comply with the Statistics Policy: https://jp.msubmit.net/cgi-bin/main.plex?form_type=display_requirements#statistics.

In summary:

- If n {less than or equal to} 30, all data points must be plotted in the figure in a way that reveals their range and distribution. A bar graph with data points overlaid, a box and whisker plot or a violin plot (preferably with data points included) are acceptable formats.

- If n > 30, then the entire raw dataset must be made available either as supporting information, or hosted on a not-for-profit repository, e.g. FigShare, with access details provided in the manuscript.

- 'n' clearly defined (e.g. x cells from y slices in z animals) in the Methods. Authors should be mindful of pseudoreplication.

- All relevant 'n' values must be clearly stated in the main text, figures and tables.

- The most appropriate summary statistic (e.g. mean or median and standard deviation) must be used. Standard Error of the Mean (SEM) alone is not permitted.

- Exact p values must be stated. Authors must not use 'greater than' or 'less than'. Exact p values must be stated to three significant figures even when 'no statistical significance' is claimed.

- Please include an Abstract Figure file, as well as the Figure Legend text within the main article file. The Abstract Figure is a piece of artwork designed to give readers an immediate understanding of the research and should summarise the main conclusions. If possible, the image should be easily 'readable' from left to right or top to bottom. It should show the physiological relevance of the manuscript so readers can assess the importance and content of its findings. Abstract Figures should not merely recapitulate other figures in the manuscript. Please try to keep the diagram as simple as possible and without superfluous information that may distract from the main conclusion(s). Abstract Figures must be provided by authors no later than the revised manuscript stage and should be uploaded as a separate file during online submission labelled as File Type 'Abstract Figure'. Please also ensure that you include the figure legend in the main article file. All Abstract Figures should be created using BioRender. Authors should use The Journal's premium BioRender account to export high-resolution images. Details on how to use and access the premium account are included as part of this email.

EDITOR COMMENTS

Reviewing Editor: Methods Details:
Methods should include the origin and strain of mice and post-recording procedures.

Comments to the Author (Required):

Thank you for submitting your manuscript to The Journal of Physiology. The manuscript was seen by two reviewers who are generally find that the (somewhat subtle) effects of noise on cells of the superior paraolivary nucleus are interesting and valuable. However, both reviewers also raise several important issues which require attention. One reviewer raises substantial concerns on the statistical data analysis, for example on the use of the number of cells instead of the number of animals as "n". The second reviewer asked for changes in data presentation, discussion and interpretation. Details on animal strain and post-recording proceedings should also be given.

Please also see 'Required Items' above.

Senior Editor:

Comments for Authors to ensure the paper complies with the Statistics Policy (Required):
Thank you for providing all data points in the histograms, but please ensure normally distributed data are presented as mean {plus minus} SD, not mean {plus minus} SEM, throughout the text

Comments to the Author:
Thank you for submitting your manuscript to The Journal of Physiology. I have now received comments from two independent reviewers and a reviewing editor, all experts in the field. All see merit in your work, but raise concerns regarding how the data are presented. I now invite you to address the reviewers' concerns and provide detailed responses to each comment. I look forward to receiving your revised manuscript in due course.

REFEREE COMMENTS

Referee #1:

The paper "Noise-induced reduction and early recovery of SPN OFF responses" by Stancu et al examines changes in SPN responses after noise exposure, including changes in intrinsic properties and inhibition. It is important to document the consequences of traumatic noise exposure in higher auditory areas, to understand how tinnitus and hyperacusis arise, so

the data are valuable. The work is being done by experts in the field, so there is high confidence in the quality of the experiments and analysis. The multiplicity of approaches is to be commended, as it rounds out a more complete picture of the effects of noise exposure. There are some confusing things about the specific findings presented in Figures 4-7, related to how the experimental results are interpreted. The most important arises in the last figure, which addresses the response properties of SPN neurons after noise exposure. It seems to mainly come down to thresholds that are elevated by about the same amount as the threshold shift induced in the periphery by noise exposure. Otherwise, responses show few differences, so the consequences of changes in inhibition and input resistance shown earlier aren't very clear. This can come across as a negative result, but a thorough and hard-won one.

Major comments:

* Fig. 2AB: The division of SPN neurons into different rate classes is interesting. For the purposes of panel B, it would be valuable to show the complete distribution from each time point, e.g. as a histogram or cumulative frequency plot. Changes in the distribution of spontaneous rates are described at 24 hours after noise exposure, but it is hard to tell if this is meaningful. This can be tested statistically, such as by comparing the firing rate distributions using Mann-Whitney or Kolmogorov-Smirnov tests.

* Fig. 4: The mIPSC frequency increases immediately after noise exposure, which is a striking finding. It would be good to mark the detected mIPSCs in panel A, which are hard to discern from background noise. Also, it would be valuable to show those detected mIPSCs overlaid at right (in place of the single control example). Furthermore, it would be valuable to show a histogram of mIPSC amplitudes compared to the amplitude distribution of noise. The average mIPSC amplitude in C appears very close to the peak-to-peak noise in the traces in A, which means that detection is a real concern. This can lead to overestimation of amplitude and underestimation of frequency, so it will reassure the reader to show more steps in the analysis.

Related to this, the abstract comments that elevated mIPSC frequency is corroborated by anatomical changes (line 55). However, mIPSC frequency increases at 0 hr post noise exposure, even though there aren't anatomical differences observed at that time (Fig. 5E). That suggests that changes in mIPSC frequency do not require anatomical changes, though of course those could also contribute. A comment about this would be valuable.

In addition, line 302 states that there is a "further elevation" in mIPSC frequency at 24 hr compared to 0 hr. However, there is no statistical difference between these groups in 4B ($P = 1$). It isn't clear why the statistical result is not to be trusted.

* Figure 5: This figure contains a central finding of the paper, suggesting that inhibitory puncta increase 24 hr after noise exposure. As shown in panel F, the density of gephyrin- and GlyT2-positive puncta 24 hrs after noise exposure are greater in contralateral SPN than ipsilateral (control) SPN. However, those values on the contralateral side in panel F appear similar to the values of both ipsi (control) and contra SPN 0 hrs post noise in panel E. That means that another interpretation is that the ipsilateral density decreases 24 hrs post noise in F. Is there a reason to dismiss the effect as an ipsilateral decrease? Or can this visual impression be resolved by comparing data from 0 hrs and 24 hrs statistically?

* Figure 6 # inputs vs boutons: The analysis in Figure 6 addresses the number of synaptic inputs to SPN neurons in two different ways. One is ramping up the stimulation amplitude and counting the number of steps of increases in EPSC amplitude, which comes out to ~4 with or without noise exposure. The second approach takes the ratio of the maximal EPSC divided by the minimal EPSC, which surprisingly yields much larger values, closer to 10 or 15. One might have thought that the minimal EPSC represented the amplitude of a single synaptic input, and the maximal EPSC represented the summed amplitude of all the synaptic inputs, yet that doesn't seem to be the case. It would be helpful to clarify what is thought to be happening here. Why does the second approach yield a different number from the first, and why is the value from the second approach referred to as "boutons" (rather than "inputs") ? The analysis in panel 6E divides the number of inputs from panel C by the number of boutons in panel D. Please clarify the logic for how this would represent the number of boutons per MNTB input. Perhaps this would be clearer with a statement of what the minimal EPSC is thought to represent. Is it the presynaptic axon with the lowest threshold, or is it a portion of an axonal arbor, that is somehow stimulated without recruiting a full axon?

* Figure 7 in vivo responses: This is a very valuable figure, that helps to illuminate how the cellular changes described so far may influence function. The ABR data of Figure 1 suggest that thresholds in the cochlea shift by 40 dB, which seems to account for most of the differences in panels E-G and I-L of Figure 7, and these analyses could therefore be viewed as red herrings. Once differences in threshold are taken into account (in panels M-P), there are few remaining differences in response. The analysis in panel G should also be repeated with the difference in thresholds taken into consideration. The major surviving difference is spontaneous rate (7H), and it is interesting to consider how increased excitability in SPN could contribute. The take-away seems to be that the SPN retains similar function despite the cellular changes identified in Figs. 2-

6, but one can't help wondering what purpose the cellular changes serve. They are described as helping "mediate early recovery" (key point #5) and serving "to recover sound-offset responses" (line 57), but it isn't clear what is being recovered from, nor what is early about it. Perhaps these ideas could be better explained.

Minor comments

line 63: "Sensory neurons" should probably be specifically "Auditory neurons". (Many sensory neurons do not encode auditory stimuli.)

line 164: "150- 200-m thick": It should be "µm".

lines 187ff: Please clarify the resolution of intensity and frequency measurements for the tuning curves of Fig. 7AB in the methods. It is difficult to estimate from the plots, which appear smoothed.

line 220: Please describe what is known about the consequences of such levels of noise exposure. Would this kind of exposure induce permanent threshold shifts or temporary threshold shifts? Does this cause IHC and OHC damage, or auditory nerve synaptopathy? Referring to others' findings would be adequate.

line 236: The short mention of spontaneous activity is confusing here. The phenomenon explored by Tritsch et al ends after degradation of Kolliker's organ around the onset of hearing (~P14 in mice). Mouse ages were only specified for the slice experiments, but it is supposed that the rest are all after the onset of hearing. Spontaneous activity does persist after the onset of hearing, presumably driven by hair cells not Kolliker's organ. It seems a different reference about spontaneous activity would be more appropriate here, e.g. from Glowatzki's opus?

Figure 2: The orientations of panel D & I are counter-intuitive. Normally, depolarized values are plotted as higher, either upwards or rightwards. The more depolarized 24 hr bar in D is lower, not higher, even though the cells are likely more excitable. The V1/2 activates at more negative potentials, but the bar is higher. Perhaps the left axes should be inverted. Please fix the labelling in panels H (zero is labelled "1.0") and I (V1/2 should have negative values). Please check the statistical outcomes in D and E (and 3CD, 4BC, 5B). The notation "P {greater than or equal to} 1.000" is confusing: P can't be greater than 1 and obtaining P = 1 in a statistical test also seems unlikely, because it implies certainty.

Line 287: "Fig 3E-G" should probably be "Fig. 3D". Please add specific references to panels B and C in the preceding exposition. The disconnect between avg {plus minus} SEM plotted in the figures vs. the median and interquartile ranges listed in the Results gets confusing. Consider plotting median and interquartile ranges in the figures instead.

Throughout: Please state the number of cells and mice at statistical comparisons. Some figures do include some of this.

Fig. 5: Panels E and F should be "gephyrin", not "gephryn". The left axis is labelled "events/µm", but the Results text refers to "puncta/µm". Please pick one ("puncta" seems more appropriate).

Referee #2:

The authors present work on the effects of noise exposure on neurons of the superior paraolivary nucleus (SPN) that have not been investigated thus are original. Interestingly they find that some aspects are consistent with effects on other brainstem neuron types, but others that are counter that suggest tuning of central gain mechanisms in a cell-type-specific way that preserves the circuit function, thus would be expected to be impactful. The authors examine this topic using a variety of methods to assess the effect from relevant angles. The writing and presentation are generally of good quality/clarity. Major issues with analysis methods discussed in comments impact the interpretability of the work.

See attached file.

END OF COMMENTS

Review of JP-RP-2025-289987

Title: Noise-Induced Reduction and Early Recovery of SPN OFF Responses
Authors: Mihai Stancu, Ezhilarsam Rajaram, Joseph Kroeger, Benedikt Grothe, Conny Kopp-Scheinpflug

Summary
The authors present work on the effects of noise exposure on neurons of the superior paraolivary nucleus (SPN) that have not been investigated thus are original. Interestingly they find that some aspects are consistent with effects on other brainstem neuron types, but others that are counter that suggest tuning of central gain mechanisms in a cell-type-specific way that preserves the circuit function, thus would be expected to be impactful. The authors examine this topic using a variety of methods to assess the effect from relevant angles. The writing and presentation are generally of good quality/clarity. Major issues with analysis methods discussed below impact the interpretability of the work.

Major comments

1. Throughout the manuscript the data analysis suffers from pseudoreplication. The treatment group for noise exposure is an animal, however, cells are used for "n." This misrepresents cells as independent replicates which artificially inflates the power and increases the type I error rate (false positive). There are several alternative strategies to overcome pseudoreplication. The simplest is to average all cells for an animal and use n=animals. This is an option if sufficient animals were used, which is not clear in all cases. Another option to explore is the use of linear mixed models which take into account the variability within an animal and the variability between animals. There are numerous recent publications on this issue that can be referenced and other methods have been explored such as Bayesian approaches and non-linear mixed models. Some relevant publications: https://pubmed.ncbi.nlm.nih.gov/20074371/, https://pubmed.ncbi.nlm.nih.gov/40414919/, https://pubmed.ncbi.nlm.nih.gov/33464305/. In the present study, for some data types as few as three animals were used which would seem to be too small determine the effects of a treatment regardless of the statistical approach used unless variability is quite low.
2. Throughout the manuscript the application of parametric vs. non-parametric statistical analyses are not well founded. Normality tests are a poor way to decide between parametric and non-parametric tests, especially for small datasets where they have low power. Such decisions should not be automated but based on the biological nature of the parameter being measured and visualization of the data with histograms or distribution curves. Visualization may reveal things like outliers or lognormal distributions that fool normality tests but should be corrected and parametric stats used. Non-parametric tests also have very little power to find differences in small data sets. Additionally, such testing for a test can lead to inconsistent application of statistics across the same type of data within a study.
3. The identity of SPN neurons for in vivo experiments are not well documented. Exact coordinates and depth should be provided. There is no mention of histological

reconstruction of electrode tip locations. Given the depth (~5 mm) and angled approach, very small differences could lead to considerable spatial errors relative to the size of the SPN. Such confirmations from the day of recordings should have been obtained. Distinguishing SPN neurons from LSO neurons seems fraught as they have overlapping input resistance range and also receive strong contralateral inhibition. The authors mention in the results that SPN neurons have distinct waveforms (line 374) but should say exactly what the differences between LSO and MNTB are as those would be the most likely off-target locations.

4. The measurement methods for miniature inhibitory synaptic events are fraught. These measurements were done by voltage clamping the cells at 0 mV with standard current clamp internal. In these relatively leaky neurons this would result in large voltage- and space-clamp errors that would lead to an underestimate of synaptic events, even somatically localized ones (Spruston, Johnston, et al, 1993). The duration of holding the cells at 0 mV is not provided, and should be, but relatively long depolarizations can lead to regulation of voltage-gated channels and endocannabinoid system activation that could conceivably differentially modulate synaptic events in different treatment conditions. There are reports of cannabinoid receptor expression at the MNTB-LSO synapse (Chi and Kandler, 2012) so it is possible in SPN. Using high-chloride cesium-based internal would eliminate these concerns.

Other comments

1. The in vivo results are not really represented in the abstract and should be as they clarify major findings, especially the sensation-level responses. This would help justify the "recovery" mentioned in the title which is currently not supported but should be.
2. Line ~147: duration and temperature of primary incubation should be provided.
3. Line 163: The average and SEM for ages used should be provided. Also, some rationale for the age range would be helpful.
4. Line 196: The sound calibration procedures are not clear and should be fleshed out including type of speakers, mic, etc.
5. Line ~187: Should state whether mice are used only once for in vivo recordings.
6. Line 200: The authors state that "SPN neurons were identified by receiving contralateral inhibition and their location lateral to the MNTB" but it is not clear how this would distinguish them from adjacent LSO neurons.
7. Line 220: Please clarify why the relatively low noise bandwidth was chosen with some references since mice are being used.
8. It would be interesting to see how ABR thresholds were affected at 24 hrs post exposure. I suspect there is a prior study that could be referred to for this information that the authors can mention.
9. Line 236: Please clarify the spontaneous firing characteristics of SPN neurons in vivo with references. Early developmental periods do not seem relevant.
10. Line 242: The results in figure 2B are stated to be "significant" but no statistical test is provided. Some parts of a whole test like one sample t test or chi squared should be provided.

11. Line 250: The approximate time after breaking into whole-cell mode for recording RMP should be provided since this can vary over the course of washout especially with modulable channels such as HCN.
12. Line 256: Fig 2C should be referenced at the end of the sentence.
13. The “n” for experiments is not consistently provided. Sometimes in results text. Sometimes on bars in figures. Sometimes not at all. Decide on a way to do it and stick with it.
14. Line 261: The authors imply that HCN channels are active at RMN but no references are provided to support this in SPN neurons.
15. Line ~261-272: The authors should provide a correlation analysis for intrinsic membrane properties. Do cells with larger Ih currents/V1/2 have lower input resistance or lower RMP? Perhaps sag potentials could be used for cells that don’t have VC data? This information would help corroborate the connection between Ih expression/modulation, Ri differences, and the noise exposure.
16. Line 279: The authors imply that all SPN neurons exhibited sustained firing, but at 500 pA in Fig. 3D there are many cells right at the bottom of the graph suggesting there is a population that does not that should be discussed.
17. Line 279: The authors imply that all SPN neurons exhibited rebound firing, but at 500 pA in Fig. 3C there are many cell right at the bottom of the graph suggesting there is a population that does not that should be discussed.
18. Line ~282 and discussion: The authors need to more explicitly explain why they think noise exposure decreases Ih but also increases rebound firing as these would tend to counter each other. If they think Ri differences dominate they should explain why. Support with a model?
19. Line 295: References should be provided for the statement.
20. Line 348-350: Please clarify the clustering method used, k-means or density-based since the referenced authors use both.
21. Line 371 and discussion: The authors note that they could not detect SPN activity immediately after noise exposure but do not mention it later in the discussion. Some speculation, or hopefully a reference, as to why this was the case should be included.
22. Line 373-377 and earlier: The authors mention that in vivo spike shapes were diagnostic for SPN neurons. Please state the features used for this analysis. Do in vivo spike shapes differ between treatment groups? Action potential kinetics should also be examined for whole-cell current-clamp data to test whether the treatment impacts other voltage gated channel systems besides Ih.
23. Line 382: How Q10 is calculated should be added to the methods.
24. Line 389: The values on the x axis of Fig. 7G (75-150) are not clearly relatable to the results provided (10-15) and should be clarified.
25. Line 475: Is this heading appropriate given the emphasis on spectral as opposed to spatial asymmetry?
26. Has OFF response preservation after sound exposure been observed in other areas, IC for instance? If so, please discuss and provide references.

27. Line 493, 515: The authors cite Parker et al 2025 and use it to support discussion topics. This is a biorxiv article which is not peer-reviewed and should not be relied upon. Such actions can mislead the field.
28. Fig 2D and more: Showing mean +/- SEM but providing medians etc. in the results section is confusing/inconsistent.
29. Fig. 2F: These experiments would have been much more convincing if additional blockers would have been added to the ACSF to provide better voltage-/space-clamp (DiAP, TEA, 4-AP, Ba, TTX, Ni, Co) as well as using ZD to block Ih in at least a subset of experiments and determine how much of the remaining current is actually attributable to Ih.
30. Fig. 2I: y axis should be negative or otherwise explained.
31. Figure 5: Quantifying sites with colocalized GlyT2 and gephyrin would be more convincing as these would be more likely to be functional synapses.
32. Figures 7I,J,M,N: The y axis needs to be explained better. # of spikes/bin width?

We thank the editors and reviewers for their time to carefully go through our manuscript and to provide us with such detailed feedback. We addressed each point and notably improved our manuscript. Main changes:

1. Statistics in figures 1-6 are now performed on the level of mice, averaging all cells per mouse
2. Statistics for in vivo data in figures 7-8 are performed using linear mixed models
3. Additional experiments were performed to add the 24hrs time point to the ABR recordings in figure 1
4. New analysis was performed to include action potential half-width for all three conditions
5. Additional *in vivo* experiments were performed to demonstrate the absence of OFF responses at 0hrs.

Please find our detailed point-by-point responses to in blue and *italic* below.

REVIEWING EDITOR COMMENTS:

Reviewing Editor: Methods Details:

Methods should include the origin and strain of mice and post-recording procedures.

Both pieces of information are now added to the methods.

Comments to the Author (Required):

Thank you for submitting your manuscript to The Journal of Physiology. The manuscript was seen by two reviewers who are generally find that the (somewhat subtle) effects of noise on cells of the superior paraolivary nucleus are interesting and valuable. However, both reviewers also raise several important issues which require attention. One reviewer raises substantial concerns on the statistical data analysis, for example on the use of the number of cells instead of the number of animals as "n". The second reviewer asked for changes in data presentation, discussion and interpretation. Details on animal strain and post-recording proceedings should also be given.

Statistics are now performed with N as the number of mice, averaging the data of individual neurons per mouse. Details on animal strain and post-recording procedures are added to the methods.

Senior Editor COMMENTS:

Comments for Authors to ensure the paper complies with the Statistics Policy (Required):

Thank you for providing all data points in the histograms, but please ensure normally distributed data are presented as mean {plus minus} SD, not mean {plus minus} SEM, throughout the text

Normally distributed data are now presented as mean {plus minus} SD. To comply with reviewer 2, we now averaged all single cell data per animal and performed the statistics on the level of animals. According to the Journal of Physiology's guideline, we additionally show single cell data as dots if the sample size is at or below 30 and N=mouse data as dots if recorded cell numbers exceed 30. When single cell data are shown, they are color-coded according to the animal they were recorded from.

Comments to the Author:

Thank you for submitting your manuscript to The Journal of Physiology. I have now received comments from two independent reviewers and a reviewing editor, all experts in the field. All see merit in your work, but raise concerns regarding how the data are presented. I now invite you to address the reviewers' concerns and provide detailed responses to each comment. I look forward to receiving your revised manuscript in due course.

REFEREE COMMENTS

Referee #1:

The paper "Noise-induced reduction and early recovery of SPN OFF responses" by Stancu et al examines changes in SPN responses after noise exposure, including changes in intrinsic properties and inhibition. It is important to document the consequences of traumatic noise exposure in higher auditory areas, to understand how tinnitus and hyperacusis arise, so the data are valuable. The work is being done by experts in the field, so there is high confidence in the quality of the experiments and analysis. The multiplicity of approaches is to be commended, as it rounds out a more complete picture of the effects of noise exposure. There are some confusing things about the specific findings presented in Figures 4-7, related to how the experimental results are interpreted. The most important arises in the last figure, which addresses the response properties of SPN neurons after noise exposure. It seems to mainly come down to thresholds that are elevated by about the same amount as the threshold shift induced in the periphery by noise exposure. Otherwise, responses show few differences, so the consequences of changes in inhibition and input resistance shown earlier aren't very clear. This can come across as a negative result, but a thorough and hard-won one.

Thank you for your commendation!!

Major comments:

* Fig. 2AB: The division of SPN neurons into different rate classes is interesting. For the purposes of panel B, it would be valuable to show the complete distribution from each time point, e.g. as a histogram or cumulative frequency plot. Changes in the distribution of spontaneous rates are described at 24 hours after noise exposure, but it is hard to tell if this is meaningful. This can be tested statistically, such as by comparing the firing rate distributions using Mann-Whitney or Kolmogorov-Smirnov tests.

We now show only two representative examples of either silent or spontaneously active neurons. The pie charts illustrate that a greater proportion of neurons are spontaneously active following noise exposure. As suggested, we also present the full distribution of spontaneous firing rates at each time point as cumulative distributions with N=number of mice (data averaged per animal as requested by reviewer 2). At the 24-hour time point, the distribution was significantly shifted toward higher spontaneous rates ($p = 0.038$; Kruskal–Wallis ANOVA with Dunn post hoc tests).

* Fig. 4: The mIPSC frequency increases immediately after noise exposure, which is a striking finding. It would be good to mark the detected mIPSCs in panel A, which are hard to discern from background noise. Also, it would be valuable to show those detected mIPSCs overlaid at right (in place of the single control example). Furthermore, it would be valuable to show a histogram of mIPSC amplitudes compared to the amplitude distribution of noise. The average mIPSC amplitude in C appears very close to the peak-to-peak noise in the traces in A, which means that detection is a real concern. This can lead to overestimation of amplitude and underestimation of frequency, so it will reassure the reader to show more steps in the analysis.

We now present longer (10 s) example recordings in which each detected mIPSC is marked by a red dot. Overlays and average mIPSC waveforms corresponding to each example are shown alongside the recorded traces. The averaged mIPSCs exhibit a steep rising phase followed by an exponential decay, consistent with postsynaptic

currents. We also plot the peak-to-peak amplitudes of the electrical noise, which show only minimal overlap with detected mIPSCs.

Related to this, the abstract comments that elevated mIPSC frequency is corroborated by anatomical changes (line 55). However, mIPSC frequency increases at 0 hr post noise exposure, even though there aren't anatomical differences observed at that time (Fig. 5E). That suggests that changes in mIPSC frequency do not require anatomical changes, though of course those could also contribute. A comment about this would be valuable.

Complying with reviewer 2, statistical analyses now include outlier analyses and are based on data averaged per mouse; accordingly, increases in mIPSC frequency and amplitude reach significance only at 24 hours. These results are now also consistent with the immunolabeling data in Fig. 5.

In addition, line 302 states that there is a "further elevation" in mIPSC frequency at 24 hr compared to 0 hr. However, there is no statistical difference between these groups in 4B (P = 1). It isn't clear why the statistical result is not to be trusted.

This has been corrected according to the statistical result.

* Figure 5: This figure contains a central finding of the paper, suggesting that inhibitory puncta increase 24 hr after noise exposure. As shown in panel F, the density of gephyrin- and GlyT2-positive puncta 24 hrs after noise exposure are greater in contralateral SPN than ipsilateral (control) SPN. However, those values on the contralateral side in panel F appear similar to the values of both ipsi (control) and contra SPN 0 hrs post noise in panel E. That means that another interpretation is that the ipsilateral density decreases 24 hrs post noise in F. Is there a reason to dismiss the effect as an ipsilateral decrease? Or can this visual impression be resolved by comparing data from 0 hrs and 24 hrs statistically?

To rigorously evaluate whether inhibitory markers are reduced ipsilateral to noise exposure between 0 and 24 hours, we conducted an ANOVA with Holm-Sidak all-pairwise multiple-comparison post hoc testing across the four gephyrin groups and, independently, across the four GlyT2 groups. This analysis revealed no significant differences between the ipsilateral 0-hour and 24-hour conditions for either gephyrin (p = 0.187) or GlyT2 (p = 0.122), indicating no detectable time-dependent reduction in inhibitory marker expression over this interval. We included this analysis in the manuscript.
All results for 30 ipsilateral neurons are now averaged per animal and compared against the average of the 30 contralateral neurons in the same animal. To demonstrate the changes within each animal we included lines connecting the means of the ipsi and contra data for each animal in the group.

* Figure 6 # inputs vs boutons: The analysis in Figure 6 addresses the number of synaptic inputs to SPN neurons in two different ways. One is ramping up the stimulation amplitude and counting the number of steps of increases in EPSC amplitude, which comes out to ~4 with or without noise exposure. The second approach takes the ratio of the maximal EPSC divided by the minimal EPSC, which surprisingly yields much larger values, closer to 10 or 15. One might have thought that the minimal EPSC represented the amplitude of a single synaptic input, and the maximal EPSC represented the summed amplitude of all the synaptic inputs, yet that doesn't seem to be the case. It would be helpful to clarify what is thought to be happening here. Why does the second approach yield a different

number from the first, and why is the value from the second approach referred to as "boutons" (rather than "inputs") ? The analysis in panel 6E divides the number of inputs from panel C by the number of boutons in panel D. Please clarify the logic for how this would represent the number of boutons per MNTB input. Perhaps this would be clearer with a statement of what the minimal EPSC is thought to represent. Is it the presynaptic axon with the lowest threshold, or is it a portion of an axonal arbor, that is somehow stimulated without recruiting a full axon?

We apologize for any lack of clarity in our original description. In these experiments, the MNTB ipsilateral to the SPN was stimulated with progressively increasing stimulus intensities, eliciting IPSCs of varying amplitudes (Fig. 6A). While discrete IPSC amplitude steps are visually apparent, these differences were quantitatively evaluated using cluster analysis. This analysis consistently identified four distinct IPSC amplitude clusters, which we interpret as reflecting convergent input from four individual MNTB neurons onto a single SPN neuron.
This interpretation is supported by our previous work combining sparse biocytin labeling of individual MNTB neurons (one neuron per slice) with GlyT2 immunostaining, which demonstrated that approximately 25% of GlyT2-positive boutons contacting SPN neurons were co-labeled with biocytin (Rajaram et al., 2020). In the same study, biocytin labeling revealed that each MNTB axon forms multiple synaptic boutons on its target SPN neuron. Physiologically, this bouton number corresponds to the observed maximum divided by minimum IPSC amplitudes. By relating the total number of boutons to the number of converging input fibers, we can estimate the number of boutons contributed by each afferent MNTB neuron.
The mechanism underlying the incomplete recruitment of all boutons during afferent stimulation remains unresolved and will be addressed in future studies. Following noise exposure, we observe an expansion of the MNTB axonal arbor and the formation of additional synaptic boutons on SPN neurons, while the number of afferent MNTB neurons remains unchanged. To clarify this structural–functional relationship, we have reorganized the figure and added a schematic illustration (new Fig. 6F).

* Figure 7 in vivo responses: This is a very valuable figure, that helps to illuminate how the cellular changes described so far may influence function. The ABR data of Figure 1 suggest that thresholds in the cochlea shift by 40 dB, which seems to account for most of the differences in panels E-G and I-L of Figure 7, and these analyses could therefore be viewed as red herrings. Once differences in threshold are taken into account (in panels M-P), there are few remaining differences in response. The analysis in panel G should also be repeated with the difference in thresholds taken into consideration. The major surviving difference is spontaneous rate (7H), and it is interesting to consider how increased excitability in SPN could contribute. The take-away seems to be that the SPN retains similar function despite the cellular changes identified in Figs. 2-6, but one can't help wondering what purpose the cellular changes serve. They are described as helping "mediate early recovery" (key point #5) and serving "to recover sound-offset responses" (line 57), but it isn't clear what is being recovered from, nor what is early about it. Perhaps these ideas could be better explained.

We apologize for the lack of clarity in the original description. In the initial submission, recordings were obtained from separate groups of mice in the control, 0hrs, or 24hrs conditions. At 0hrs post-exposure, we recorded from spontaneously active neurons with characteristic SPN waveforms; however, no OFF responses could be evoked, even at the highest stimulus intensities.
During the revision phase, we performed additional experiments in which SPN OFF neurons were recorded before noise exposure, followed by a 2-h noise exposure while maintaining the electrode position. Recordings then continued from the same SPN location (though not necessarily the same cell) that exhibited OFF responses prior to

exposure. Despite ongoing spontaneous activity indicating viable neurons, OFF responses were again absent. An example of this experiment is shown in revised Fig. 7B.

We therefore hypothesize that the period up to 24hrs post-exposure is required for changes in excitability and inhibition that enable recovery of OFF responses. Notably, although peripheral thresholds had not recovered by 24hrs, OFF responses in the SPN were restored, and when corrected for threshold shifts, were even comparable to pre-exposure levels. This interpretation is now explicitly described in the revised manuscript.

Minor comments

line 63: "Sensory neurons" should probably be specifically "Auditory neurons". (Many sensory neurons do not encode auditory stimuli.)

Changed as suggested.

line 164: "150- 200-m thick": It should be "µm".

Corrected – thank you for catching this!

lines 187ff: Please clarify the resolution of intensity and frequency measurements for the tuning curves of Fig. 7AB in the methods. It is difficult to estimate from the plots, which appear smoothed.

This information has been added to the figure legend: Tuning curves for each recorded cell were measured using pure tones centered on a cell-specific reference frequency and spanning ~1.5 octaves above and below, with at least 10 frequency steps. Sound intensity was varied in 5 dB SPL increments. Tuning curves were smoothed using modified Akima piecewise-cubic interpolation on a 1000 × 1000 grid.

line 220: Please describe what is known about the consequences of such levels of noise exposure. Would this kind of exposure induce permanent threshold shifts or temporary threshold shifts? Does this cause IHC and OHC damage, or auditory nerve synaptopathy? Referring to others' findings would be adequate.

Yes, this noise exposure paradigm is known to induce synaptopathy at the inner hair cells although ABR threshold recover. We included this in the discussion.

line 236: The short mention of spontaneous activity is confusing here. The phenomenon explored by Tritsch et al ends after degradation of Kolliker's organ around the onset of hearing (~P14 in mice). Mouse ages were only specified for the slice experiments, but it is supposed that the rest are all after the onset of hearing. Spontaneous activity does persist after the onset of hearing, presumably driven by hair cells not Kolliker's organ. It seems a different reference about spontaneous activity would be more appropriate here, e.g. from Glowatzki's opus?

We strongly agree with the reviewer that spontaneous activity persists after the onset of hearing. However, the point we intended to make is that, in brain slice preparations, auditory brainstem neurons are disconnected from inner hair cells (IHCs) and therefore lose their primary source of spontaneous firing. Unlike neurons such as Purkinje cells, the intrinsic membrane properties of auditory brainstem neurons do not, in general, support spontaneous action potential generation. We corrected our statement accordingly and also included the valuable work of the Glowatzki lab showing "a decline of intrinsic firing in ANFs during posthearing maturation".

Figure 2: The orientations of panel D & I are counter-intuitive. Normally, depolarized values are plotted as higher, either upwards or rightwards. The more depolarized 24 hr bar in D is lower, not higher, even though the cells are likely more excitable. The V1/2 activates at more negative potentials, but the bar is higher. Perhaps the left axes should be inverted. Please fix the labelling in panels H (zero is labelled "1.0") and I (V1/2 should have negative values). Please check the statistical outcomes in D and E (and 3CD, 4BC, 5B). The notation "P {greater than or equal to} 1.000" is confusing: P can't be greater than 1 and obtaining P = 1 in a statistical test also seems unlikely, because it implies certainty.

We are grateful for these helpful suggestions. We have inverted and corrected the axes in question. The statistics were all re-run on the level of mice (neurons for each mouse were averaged) to comply with reviewer 2. P-values were corrected as suggested. We decided to remove the I_h data from the manuscript, as without many more additional experiments on changes in potassium currents these results remain inconclusive.

Line 287: "Fig 3E-G" should probably be "Fig. 3D". Please add specific references to panels B and C in the preceding exposition. The disconnect between avg {plus minus} SEM plotted in the figures vs. the median and interquartile ranges listed in the Results gets confusing. Consider plotting median and interquartile ranges in the figures instead. Throughout: Please state the number of cells and mice at statistical comparisons. Some figures do include some of this.

Reference to figures have been corrected. Since statistics were all re-run on the level of mice (neurons for each mouse were averaged) to comply with reviewer 2, firing rates per mouse follow normal distributions and are plotted as mean ±SD and also referred to as such in the text. We now also state throughout the text the number of cells and number of animals.

Fig. 5: Panels E and F should be "gephyrin", not "gephryn". The left axis is labelled "events/µm", but the Results text refers to "puncta/µm". Please pick one ("puncta" seems more appropriate).

Corrected – thank you for spotting this!

Referee #2:

The authors present work on the effects of noise exposure on neurons of the superior paraolivary nucleus (SPN) that have not been investigated thus are original. Interestingly they find that some aspects are consistent with effects on other brainstem neuron types, but others that are counter that suggest tuning of central gain mechanisms in a cell-type-specific way that preserves the circuit function, thus would be expected to be impactful. The authors examine this topic using a variety of methods to assess the effect from relevant angles. The writing and presentation are generally of good quality/clarity. Major issues with analysis methods discussed in comments impact the interpretability of the work.

Major comments

1. Throughout the manuscript the data analysis suffers from pseudoreplication. The treatment group for noise exposure is an animal, however, cells are used for "n." This misrepresents cells as independent replicates which artificially inflates the power and increases the type I error rate (false positive). There are several alternative strategies to overcome pseudoreplication. The simplest is to average all cells for an animal and use n=animals. This is an option if sufficient animals were used, which is not clear in all cases. Another option to explore is the use of linear mixed models which take into account the variability within an animal and the variability between animals. There are numerous recent publications on this issue that can be referenced and other methods have been explored such as Bayesian approaches and non-linear mixed models.

Some relevant publications:
https://pubmed.ncbi.nlm.nih.gov/20074371/,
https://pubmed.ncbi.nlm.nih.gov/40414919/, https://pubmed.ncbi.nlm.nih.gov/33464305/.

To comply with this reviewer all statistics in the manuscript are now performed on the level of mice. In accordance with the Journal of Physiology's policy we nevertheless provide the data for individual cells as dots in the graphs (color-coded by the mouse the data originate from). When individual cell number were more than n=30, the dots in the figures represent the number of mice.

In the present study, for some data types as few as three animals were used which would seem to be too small determine the effects of a treatment regardless of the statistical approach used unless variability is quite low.

We now report (n) as the number of neurons and (N) as the number of mice in the figure legends and text throughout the manuscript. The statistics are now performed with number of mice as N. However, showing the individual neurons for each animal has value in the community and therefore we keep presenting the individual data points. They are now color-coded by the mice the data originate from. The only experiment were an N = 3 animals was used is the immunolabeling experiment were the statistical power is larger as it is a paired comparison within each animal (Fig.5).

2. Throughout the manuscript the application of parametric vs. non-parametric statistical analyses are not well founded. Normality tests are a poor way to decide between parametric and non-parametric tests, especially for small datasets where they have low power. Such decisions should not be automated but based on the biological nature of the parameter being measured and visualization of the data with histograms or distribution curves. Visualization may reveal things like outliers or lognormal distributions that fool normality tests but should be

corrected and parametric stats used. Non-parametric tests also have very little power to find differences in small data sets. Additionally, such testing for a test can lead to inconsistent application of statistics across the same type of data within a study.

To address the reviewers' suggestion, we now included the IQR outlier detection test for each of our data sets before averaging the neurons per mouse and performing the normality test. We also plot individual data points, which allows for visual inspection of the data. Then, for normally distributed data we perform t-test when 2 groups are compared and ANOVAs followed by all pairwise Multiple Comparison Procedures (Holm–Sidak post hoc), when 3 groups are compared. When the normality assumption was violated, nonparametric tests (Mann–Whitney U test for comparing two groups and Kruskal–Wallis ANOVA on ranks for comparing three or more groups) were used. Outlier detection/removal and averaging data per mouse mostly smoothed the data and made results easier to interpret.

3. The identity of SPN neurons for in vivo experiments are not well documented. Exact coordinates and depth should be provided. There is no mention of histological reconstruction of electrode tip locations. Given the depth (~5 mm) and angled approach, very small differences could lead to considerable spatial errors relative to the size of the SPN. Such confirmations from the day of recordings should have been obtained. Distinguishing SPN neurons from LSO neurons seems fraught as they have overlapping input resistance range and also receive strong contralateral inhibition. The authors mention in the results that SPN neurons have distinct waveforms (line 374) but should say exactly what the differences between LSO and MNTB are as those would be the most likely off-target locations.

We identify SPN neurons by a 3-4 step procedure:

1. *During the initial calibration of our stereotaxic frame a couple of years ago, ink injections were used to confirm each recording site in subsequent histological reconstructions.*
2. *Since identifying a range of coordinates with respect to Bregma, the SPN is targeted so reliably that reconstructions are not done on a regular basis anymore. Our set of anterior-posterior and lateral coordinate ranges with respect to Bregma as well as the depth are now provided in the methods. As you will see we enter the brain at a lateral location in line with the SPN – mostly avoiding any angles of the electrode.*
3. *When targeting SPN cells, we start out from finding the lateral edge of the MNTB (identified by responding to contralateral stimulation only and the prepotentials in the waveform) and then move lateral from there. During the subsequent search for SPN neurons we always test contralateral **and** ipsilateral responsiveness. While it is correct that LSO neurons also receive strong contralateral inhibition, they also receive strong ipsilateral excitation, which SPN neurons do not.*
4. *The extracellular waveform of SPN neurons is so different from MNTB and LSO, that this is another good marker for SPN neurons. We provide an example each of the three nuclei for the reviewer to see here. In the manuscript we are now showing SPN waveforms in a designated panel and point the reader towards published waveforms of MNTB and LSO recorded under nearly identical experimental conditions.*

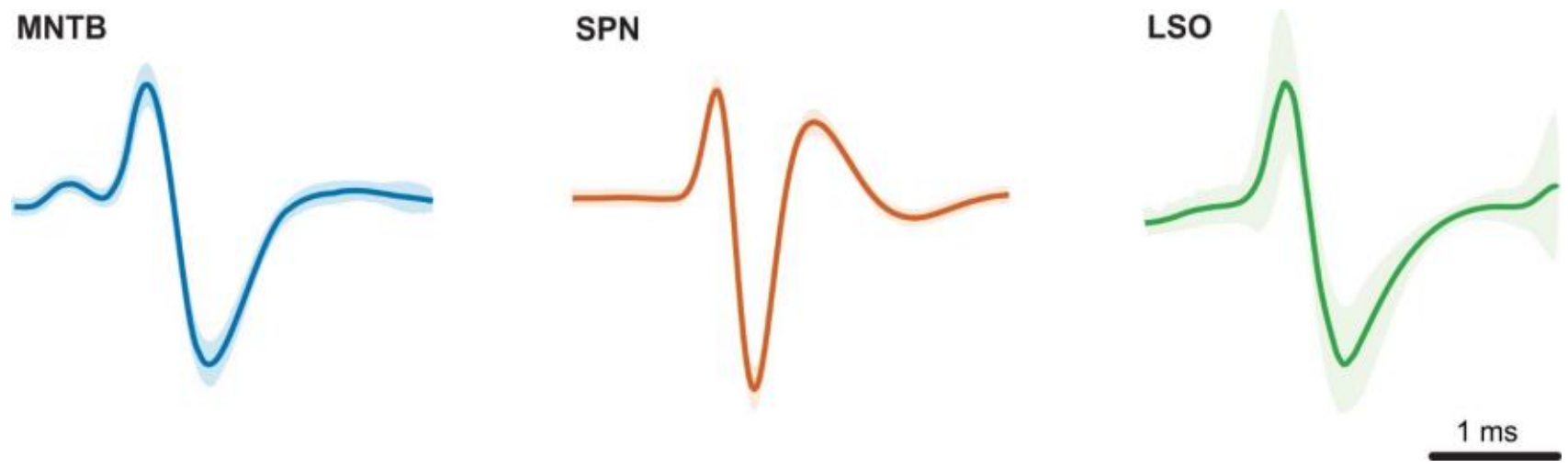

4. The measurement methods for miniature inhibitory synaptic events are fraught. These measurements were done by voltage clamping the cells at 0 mV with standard current clamp internal. In these relatively leaky neurons this would result in large voltage- and space-clamp errors that would lead to an underestimate of synaptic events, even somatically localized ones (Spruston, Johnston, et al, 1993). The duration of holding the cells at 0 mV is not provided, and should be, but relatively long depolarizations can lead to regulation of voltage-gated channels and endocannabinoid system activation that could conceivably differentially modulate synaptic events in different treatment conditions. There are reports of cannabinoid receptor expression at the MNTB-LSO synapse (Chi and Kandler, 2012) so it is possible in SPN. Using high-chloride cesium-based internal would eliminate these concerns.

We agree that space-clamp errors are an inherent caveat when voltage clamping large neurons with extensive dendritic arbors. However, since glycinergic synapses on SPN neurons have been shown to be located predominantly at the soma, space clamp errors will be small as suggested by Spruston et al, 1993. In case of potential underestimation of synaptic events due to these measurement methods, it would equally affect the experimental groups and should not change the results between groups.
Thank you for the suggestion of using high-chloride cesium-based internal solution in the future. However, in our current experiments, neurons were held at 0mV for very short time scales of 10 times 2sec sweeps and with the cell being back at its resting potential in between sweeps. Data from three mini-protocols being averaged per cell.
Agreed - modulatory effects of any neuromodulator are always possible and have to be kept in mind for the big picture. However, it is beyond the scope of our present manuscript to address this reviewers' particular concern about activating the endocannaboid system during the 2 second mIPSC recording.
Although Chi and Kandler (2012) showed the expression of tonotopically organized CB1 expression at P5 and P12 in rats, the Allen Brain Atlas (https://mouse.brain-map.org/gene/show/12584) does not show expression of CB1 in adult (P56) mice. Although this is not 100% proof that there may not be any CB1 receptor activity in the SPN nor the MNTB, it strongly reduces the possibility of engaging CB1 receptor activity during the 2sec of depolarization of mIPSC recording. Personal consultation of my colleague Dr. Lars Kuns (Trattner et al., 2013) corroborated that CB1 receptors in the rodent SOC strongly diminish following P15.

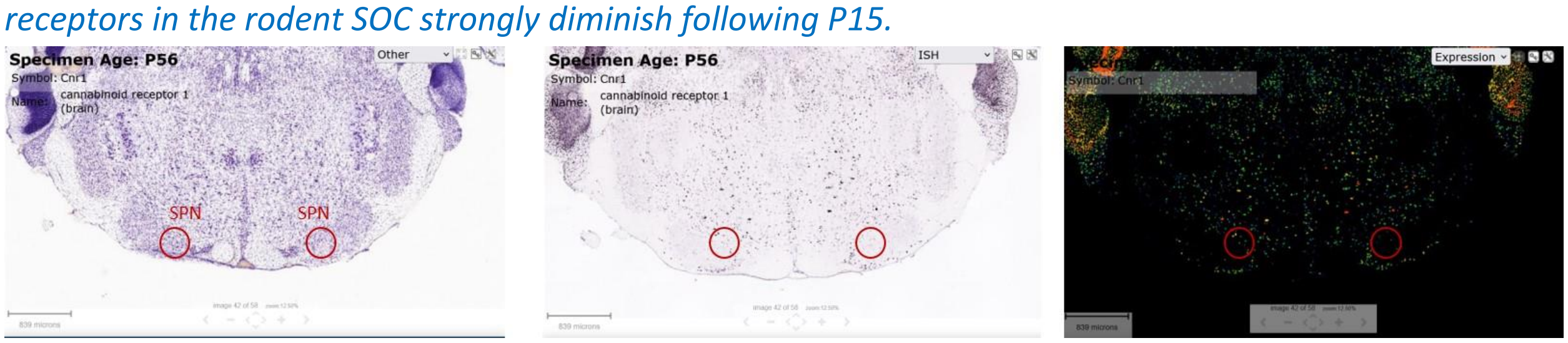

Other comments

1. The in vivo results are not really represented in the abstract and should be as they clarify major findings, especially the sensation-level responses. This would help justify the "recovery" mentioned in the title which is currently not supported but should be.

We incorporated the in vivo results in the abstract and also included more information about the lack of OFF responses at 0hrs into the results (see out response to your comment 21). This should now clarify that sound exposure abolishes SPN OFF responses immediately after exposure, but that the adaptations in intrinsic excitability and synaptic inhibition within the first 24 hours enable fast recovery of OFF responses.

2. Line ~147: duration and temperature of primary incubation should be provided.

Information has been added.

3. Line 163: The average and SEM for ages used should be provided. Also, some rationale for the age range would be helpful.

This information is now included in the methods: This post-weaning age was selected to ensure relative maturation of SPN neurons while maintaining high yield and quality in patch-clamp recordings (Rajaram et al., 2020).

4. Line 196: The sound calibration procedures are not clear and should be fleshed out including type of speakers, mic, etc.

The calibration details have been added to the methods section.

5. Line ~187: Should state whether mice are used only once for in vivo recordings.

For single-cell in vivo recordings mice were only used once. The information has been added.

6. Line 200: The authors state that "SPN neurons were identified by receiving contralateral inhibition and their location lateral to the MNTB" but it is not clear how this would distinguish them from adjacent LSO neurons.

Please also see our response to your major comment 3. We now provide the stereotaxic coordinates in the methods section and a 3-step description of how we identify SPN neurons in the results at the start of the text to figure 7.

7. Line 220: Please clarify why the relatively low noise bandwidth was chosen with some references since mice are being used.

The 8-16kHz bandwidth was used for two reasons. First to expose the mice within the range of their most sensitive hearing and second, to make the study comparable with other studies that follow this exposure paradigm introduced by the Liberman & Kujawa's laboratories {Fernandez, 2015 #3855;Bakay, 2018 #3854;Ouagazzal, 2006 #3853;Zhang, 2013 #3852;Fischl, 2019 #3679}. This information has been added to the manuscript.

8. It would be interesting to see how ABR thresholds were affected at 24 hrs post exposure. I suspect there is a prior study that could be referred to for this information that the authors can mention.

We have added data for the 24hrs time point following noise exposure to figure 1. Although there is a tendency of threshold recovery at 24 hrs – it is not significant.

9. Line 236: Please clarify the spontaneous firing characteristics of SPN neurons in vivo with references. Early developmental periods do not seem relevant.

The statement the reviewer refers to does not address spontaneous firing rates of SPN neurons in vivo. Rather it aims to explain that in the auditory system the primary source of spontaneous (not sound-evoked) activity is the cochlea. In brain slice preparations, auditory brainstem neurons are disconnected from inner hair cells (IHCs) and therefore lose their primary source of spontaneous firing. Unlike other neurons such as for example cerebellar Purkinje cells, the intrinsic membrane properties of auditory brainstem neurons do not, in general, support spontaneous action potential generation. We included more explanation and references in the text.

10. Line 242: The results in figure 2B are stated to be "significant" but no statistical test is provided. Some parts of a whole test like one sample t test or chi squared should be provided.

We now provide the spontaneous firing rates for all 3 treatment groups with N being the number of mice in each group as cumulative distribution (revised Fig. 2C). The significance was tested by Kruskal–Wallis ANOVA with Dunn post hoc tests and the 24hrs group was significantly different from the control and the 0hrs group, with no difference between control and 0hrs.

11. Line 250: The approximate time after breaking into whole-cell mode for recording RMP should be provided since this can vary over the course of washout especially with modulable channels such as HCN.

The following information has been added to the manuscript: "RMP of SPN neurons was recorded in absence of current injection within 3-10 minutes after breaking into whole-cell mode..."

12. Line 256: Fig 2C should be referenced at the end of the sentence.

Changed as requested.

13. The "n" for experiments is not consistently provided. Sometimes in results text. Sometimes on bars in figures. Sometimes not at all. Decide on a way to do it and stick with it.

We now report n = number of cells and N = number of mice in the text and in the figure legends.

14. Line 261: The authors imply that HCN channels are active at RMN but no references are provided to support this in SPN neurons.

We decided to remove the I_h data from the manuscript, as without many more additional experiments on accompanying changes in potassium currents these results remain inconclusive.

15. Line ~261-272: The authors should provide a correlation analysis for intrinsic membrane properties. Do cells with larger Ih currents/V1/2 have lower input resistance or lower RMP? Perhaps sag potentials could be used for

cells that don't have VC data? This information would help corroborate the connection between Ih expression/modulation, Ri differences, and the noise exposure.

See response to the previous comment - we decided to remove the I_h data from the manuscript, as without many more additional experiments on accompanying changes in potassium currents these results remain inconclusive.

16. Line 279: The authors imply that all SPN neurons exhibited sustained firing, but at 500 pA in Fig. 3D there are many cells right at the bottom of the graph suggesting there is a population that does not that should be discussed.

Action potential firing was elicited by current injections between -500pA and +950pA in steps of 50pA. Of the 115 neurons across the 3 experimental groups tested with these current step protocols all but 1 cell had sustained firing in response to depolarizing currents either up to +500pA or above. Although not all neurons showed sustained firing at +500pA, we chose to present this to keep the y-axis in Fig. 3B in a range where the much lower firing rates at hyperpolarizing current injections could also be visually identified. We now clarified the range of current injections in the text and included a typical example for each group depicting the average firing rates at 500pA in the revised figure 3A.

17. Line 279: The authors imply that all SPN neurons exhibited rebound firing, but at 500 pA in Fig. 3C there are many cell right at the bottom of the graph suggesting there is a population that does not that should be discussed.

The graph has changed due to your request of presenting the data per mouse rather than per cell. However, it is an important point and we included the following explanation:
Injections of −500 pA hyperpolarized the membrane to −75 to −80 mV, a range readily produced by inhibitory inputs to SPN neurons and near the threshold for rebound spike generation at stimulus offset (Kopp-Scheinpflug et al., 2011), a hallmark of SPN electrophysiology (Fig. 3A). Examining rebound spiking near threshold provides an effective measure to study increased neural gain following noise exposure. This moderate hyperpolarization elicited rebound responses in 63% (26/41) of control cells, 61% (23/38) at 0hrs, and 85% (41/48) at 24hrs post–noise exposure (Fig. 3A).

18. Line ~282 and discussion: The authors need to more explicitly explain why they think noise exposure decreases Ih but also increases rebound firing as these would tend to counter each other. If they think Ri differences dominate they should explain why. Support with a model?

We agree with the reviewer that further work is required to fully characterize the effects of noise exposure on I_H and its influence on offset responses. Accordingly, we have removed the I_H data from the current manuscript. We acknowledge that a modeling approach would provide valuable insight into these mechanisms and plan to pursue this in future studies.

19. Line 295: References should be provided for the statement.
Added as requested

20. Line 348-350: Please clarify the clustering method used, k-means or density-based since the referenced authors use both.

Similar to Rajaram et al., 2020, a density based clustering algorithm, DBSCAN (Density-Based Spatial Clustering of Applications with Noise) from scikit-learn, was used to estimate the number of inputs. A density-based approach was chosen over K-means because DBSCAN can identify clusters of arbitrary shape, whereas K-means performs well primarily with spherical clusters. Given the expected variability in both the number and strength of afferent inputs, spherical clusters were considered unlikely. We now also clarify that in the text.

21. Line 371 and discussion: The authors note that they could not detect SPN activity immediately after noise exposure but do not mention it later in the discussion. Some speculation, or hopefully a reference, as to why this was the case should be included.

Our animal welfare guidelines do not allow longitudinal single-cell in vivo recording sessions over multiple days. Therefore, upon first submission we recorded individual mice either in control, 0hrs or 24hrs condition. Although at 0hrs, we recorded from neurons with the typical SPN waveform, but no offset responses could be elicited (even at the highest intensities). We now used the revision phase to perform additional experiments were we recorded from just one SPN OFF neuron, then applied the 2-hr noise exposure, while keeping the electrode in place and then continued to record from the same location within the SPN that showed OFF responses before exposure. Again, despite spontaneous firing indicating the presence of healthy cells, no OFF responses were observed. An example of such a recording is now provided in revised figure 7B.

22. Line 373-377 and earlier: The authors mention that in vivo spike shapes were diagnostic for SPN neurons. Please state the features used for this analysis. Do in vivo spike shapes differ between treatment groups? Action potential kinetics should also be examined for whole-cell current-clamp data to test whether the treatment impacts other voltage gated channel systems besides Ih.

We now include the averaged waveforms of extracellularly recorded SPN neurons in control condition, at 0hrs and at 24hrs in Fig. 7. There is no apparent difference between the averaged spike waveforms between the three conditions. While there could be finer differences in action potential shape based on changes in sodium or potassium channel function, but we did not examine the effects of noise exposure on potassium (or sodium) channels in the present manuscript. While this represents an important and interesting avenue for future investigation, a rigorous analysis would require detailed biophysical characterization and targeted channel pharmacology. We therefore believe that addressing these questions thoroughly, rather than through a limited or preliminary analysis, is beyond the scope of the current study and is best pursued in future work.

23. Line 382: How Q10 is calculated should be added to the methods.

The calculation of Q10 is now included in the figure legend of figure 7.

24. Line 389: The values on the x axis of Fig. 7G (75-150) are not clearly relatable to the results provided (10-15) and should be clarified.

The sound is presented from 10-110ms - that is what the x-axis in previous figure 7G shows. The figure has been restructured into a new figure 8, so that latencies are presented with respect to the onset of sound only to avoid confusion.

25. Line 475: Is this heading appropriate given the emphasis on spectral as opposed to spatial asymmetry?

Thank you - corrected to "Spectral"

26. Has OFF response preservation after sound exposure been observed in other areas, IC for instance? If so, please discuss and provide references.

We included information to loss and preservation of OFF responses into the discussion.

27. Line 493, 515: The authors cite Parker et al 2025 and use it to support discussion topics. This is a biorxiv article which is not peer-reviewed and should not be relied upon. Such actions can mislead the field.

Parker et al., 2025 is now accepted for publication in the Journal of Physiology. The reference will be updated as soon as the paper is available on the JP site.

28. Fig 2D and more: Showing mean +/- SEM but providing medians etc. in the results section is confusing/inconsistent.

Corrected throughout manuscript

29. Fig. 2F: These experiments would have been much more convincing if additional blockers would have been added to the ACSF to provide better voltage-/space-clamp (DiAP, TEA, 4-AP, Ba, TTX, Ni, Co) as well as using ZD to block Ih in at least a subset of experiments and determine how much of the remaining current is actually attributable to Ih.

We agree and will include the suggestion in future experiments.

30. Fig. 2I: y axis should be negative or otherwise explained.

Yes, the y-axis should be negative – thank you for catching this!! Corrected

31. Figure 5: Quantifying sites with colocalized GlyT2 and gephyrin would be more convincing as these would be more likely to be functional synapses.

That is a good suggestion. We included this into the text.

32. Figures 7I,J,M,N: The y axis needs to be explained better. # of spikes/bin width?

PSTHs were constructed using 1 ms bins. Spike counts in each bin were converted to firing rates (spikes/s, Hz) by dividing by the bin width (1 ms) and normalizing by the number of repetitions. Therefore, y-axis is in spikes/second or Hz. This information is now given in the figure legend.

1st Revision - Editorial Decision 12-Mar-2026

Dear Dr Kopp-Scheinpflug,

Re: JP-RP-2026-289987R1 "**Noise-Induced Reduction and Early Recovery of SPN OFF Responses**" by Mihai Stancu, Ezhilarasan Rajaram, Joseph Kroeger, Benedikt Grothe, and Conny Kopp-Scheinpflug

Thank you for submitting your manuscript to The Journal of Physiology. It has been assessed by a Reviewing Editor and by 2 expert referees and we are pleased to tell you that it is acceptable for publication following satisfactory revision.

The referee reports are copied at the end of this email.

Please address all the points raised and incorporate all requested revisions or explain in your Response to Referees why a change has not been made. We hope you will find the comments helpful and that you will be able to return your revised manuscript within 4 weeks. If you require longer than this, please contact journal staff: jp@physoc.org.

Your revised manuscript should be submitted online using the link in your Author Tasks Link Not Available. This link is accessible via your account as Corresponding Author; it is not available to your co-authors. If this presents a problem, please contact journal staff (jp@physoc.org). Image files from the previous version are retained on the system. Please ensure you replace or remove any files that are being revised.

If you do not wish to submit a revised version of your manuscript, you must inform our journal staff (jp@physoc.org) or reply to this email to request withdrawal. Please note that a manuscript must be formally withdrawn from the peer review process at one journal before it may be submitted to another journal.

TRANSPARENT PEER REVIEW POLICY: To improve the transparency of its peer review process The Journal of Physiology publishes online, as supporting information, the peer review history of all articles accepted for publication. Readers will have access to decision letters, including Editors' comments and referee reports, for each version of the manuscript, as well as any author responses to peer review comments. Referees can decide whether or not they wish to be named on the peer review history document.

ABSTRACT FIGURES: Authors are expected to use The Journal's premium BioRender account to create/redraw their Abstract Figures. Information on how to access this account is here: https://physoc.onlinelibrary.wiley.com/journal/14697793/biorender-access.

This will enable Authors to create and download high-resolution figures. If authors have used the free BioRender service, they can use the instructions provided in the link above to download a high-resolution version suitable for publication.

The link provided should only be used for the purposes of this submission. Authors will be charged for figures created on this account if they are not related to this manuscript submission.

LANGUAGE EDITING AND SUPPORT FOR PUBLICATION: If you would like help with English language editing, or other article preparation support, Wiley Editing Services offers expert help, including English Language Editing, as well as translation, manuscript formatting, and figure formatting at www.wileyauthors.com/eeo/preparation. You can also find resources for Preparing Your Article for general guidance about writing and preparing your manuscript at www.wileyauthors.com/eeo/prepresources.

RIGOUR AND REPRODUCIBILITY: We recommend authors consult The Journal's Rigour and Reproducibility requirements to ensure that their manuscript is suitable for publication: https://physoc.onlinelibrary.wiley.com/pb-assets/hub-assets/physoc/documents/TJP-Rigour-and-Reproducibility-Requirements.pdf

REVISION CHECKLIST:

Check that your Methods section conforms to journal policy: https://jp.msubmit.net/cgi-bin/main.plex?form_type=display_requirements#methods

Check that data presented conforms to the statistics policy: https://jp.msubmit.net/cgi-bin/main.plex?form_type=display_requirements#statistics

Upload a full Response to Referees file. To create your 'Response to Referees' copy all the reports, including any comments from the Senior and Reviewing Editors, into a Microsoft Word, or similar, file and respond to each point, using font or background colour to distinguish comments and responses and upload as the required file type.

Please upload two versions of your manuscript text: one with all relevant changes highlighted and one clean version with no changes tracked. The manuscript file should include all tables and figure legends, but each figure/graph should be uploaded as separate, high-resolution files. The journal is now integrated with Wiley's Image Checking service. For further details, see: https://www.wiley.com/en-us/network/publishing/research-publishing/trending-stories/upholding-image-integrity-wileys-image-screening-service

You may also upload:

- 'Potential Cover Art' for consideration as the issue's cover image
- Appropriate Supporting Information (Video, audio or data set: see https://jp.msubmit.net/cgi-bin/main.plex?form_type=display_requirements#supp).

We look forward to receiving your revised submission.

If you have any queries, please reply to this email and we will be pleased to advise.

Yours sincerely,

Vaughan Macefield
Senior Editor
The Journal of Physiology

REQUIRED ITEMS

- Your manuscript must include a complete Additional Information section, including competing interests; funding; author contributions and acknowledgements.

- The Journal of Physiology funds authors of provisionally accepted papers to use the premium BioRender site to create high resolution schematic figures. Follow this link and enter your details and the manuscript number to create and download figures. Upload these as the figure files for your revised submission. If you choose not to take up this offer, we require figures to be of similar quality and resolution. If you are opting out of this service to authors, state this in the Comments section on the Detailed Information page of the submission form. The link provided should only be used for the purposes of this submission. Authors will be charged for figures created on this premium BioRender account if they are not related to this manuscript submission.

- Please include an Abstract Figure file and an Abstract Figure legend. An appropriate figure legend, which should not exceed 150 words in length, should be included in the main manuscript file. The Abstract Figure is a piece of artwork designed to give readers an immediate understanding of the research and should summarise the main conclusions. If possible, the image should be easily 'readable' from left to right or top to bottom. It should show the physiological relevance of the manuscript so readers can assess the importance and content of its findings. Abstract Figures should not merely recapitulate other figures in the manuscript. Please try to keep the diagram as simple as possible and without superfluous information that may distract from the main conclusion(s). Abstract Figures must be provided by authors no later than the revised manuscript stage and should be uploaded as a separate file during online submission labelled as File Type 'Abstract Figure'. Please also ensure that you include the figure legend in the main article file. All Abstract Figures should be created using BioRender. Authors should use The Journal's premium BioRender account to export high-resolution images. Details on how to use and access the premium account are included as part of this email.

EDITOR COMMENTS

Reviewing Editor:

Both reviewers were largely satisfied with your changes to the manuscript. However, reviewer 1 feels that your the analysis presented in Figure 6D and E allows for your conclusions and suggests to remove the two panels as Figure 5 independently supports the same conclusion. In reply to one of the reviewers, you chose to change the reported statistics from cells to animals but in the figures, data for cells is also displayed alongside data for animals. Although different shadings are used, I think that this is confusing and makes the plots hard to understand. One option, in my view, could be to show the data points for the cells in a supplementary figure but stick in the main article with the data for each animal so that it is in line with the shown statistics.

Also, a legend for the abstract figure is missing (which seemed to be a draft?) and the additional info section is missing information on competing interests, author contributions, funding and acknowledgements (if applicable).

Please see 'Required Items' above.

Senior Editor:

Thank you for submitting your revised manuscript to The Journal of Physiology. I have now received comments from two independent reviewers and a reviewing editor, all experts in the field. as you will see, the reviewers are largely satisfied with your responses, but there are some remaining issues you will need to address before we can accept your manuscript. Please note that the Journal does not accept supplementary data, so if you are to generate a new figure as per the reviewing editor's suggestion, this should be included in Results. I look forward to receiving this revised version in due course.

REFEREE COMMENTS

Referee #1:

The authors responded very thoroughly to my original comments. However, I remain confused by the assessment of "boutons" in Figure 6. It is hard to think of a mechanism whereby minimal stimulation would cause neurotransmitter release from one bouton in one input, and maximal stimulation would cause neurotransmitter release from all boutons in all inputs. Kim and Kandler (2010) (cited on line 395) seem to use the ratio of maximal to minimal stimulation to estimate the number of inputs, not boutons within single inputs. It may be advisable to avoid the assertion that one approach assesses input number and the other bouton number until that can be demonstrated. The major increase in maximal IPSC amplitude in Fig. 6C is quite convincing, and it is consistent with the increased number of boutons in Fig. 5. To my mind, the second approach in Fig. 6D&E adds confusion unless the difference between the approaches can be better explained.

Referee #2:

Review of JP-RP-2025-289987 resubmission

Title: Noise-Induced Reduction and Early Recovery of SPN OFF Responses

Authors: Mihai Stancu, Ezhilarsam Rajaram, Joseph Kroeger, Benedikt Grothe, Conny Kopp-Scheinpflug

Summary of changes and final comments

The authors have made substantial changes to the manuscript and significant effort to address the concerns raised. While the selection of parametric vs. non-parametric statistic still seems automated, the modifications made have reduced the potential impact. Figure legends should state what error bars are shown, SEM or quartiles etc. I would argue that displaying points for cells instead of animals, even with some shading differences, with error bars and stats based on animals is confusing and makes it difficult to visually interpret the graphs. This is especially true on the imaging data. Since the authors invoked journal policy on this point, I will leave this up to the editor. The loss of HCN channel information is lamentable, but the authors appear committed to finishing this as a separate project.

END OF COMMENTS

Review of JP-RP-2025-289987 resubmission

Title: Noise-Induced Reduction and Early Recovery of SPN OFF Responses
Authors: Mihai Stancu, Ezhilarsam Rajaram, Joseph Kroeger, Benedikt Grothe, Conny Kopp-Scheinpflug

Summary of changes and final comments

The authors have made substantial changes to the manuscript and significant effort to address the concerns raised. While the selection of parametric vs. non-parametric statistic still seems automated, the modifications made have reduced the potential impact. Figure legends should state what error bars are shown, SEM or quartiles etc. I would argue that displaying points for cells instead of animals, even with some shading differences, with error bars and stats based on animals is confusing and makes it difficult to visually interpret the graphs. This is especially true on the imaging data. Since the authors invoked journal policy on this point, I will leave this up to the editor. The loss of HCN channel information is lamentable, but the authors appear committed to finishing this as a separate project.

LMU
LUDWIG-
MAXIMILIANS-
UNIVERSITÄT
MÜNCHEN

FACULTY OF BIOLOGY
NEUROBIOLOGY

Prof. Dr. C. Kopp-Scheinpflug · Faculty of Biology · Großhaderner Str. 2 ·
82152 Planegg-Martinsried

The Journal of Physiology

Prof. Dr. C. Kopp-Scheinpflug

Telefon +49 (0)89 2180-74310

cks@bio.lmu.de

https://www.bio.lmu.de/en/research/research-fields/neurobiology/kopp-scheinpflug-lab/

Postanschrift:
Großhaderner Straße 2
D-82152 Planegg-Martinsried

Ihr Zeichen, Ihre Nachricht vom   Unser Zeichen

Martinsried, 23.03.2026

The Journal of Physiology Decision on Our Manuscript: "**Noise-Induced Reduction and Early Recovery of SPN OFF Responses**", JP-RP-2026-289987R2.

Dear Editors and Reviewers,

we thank the Editors for allowing us to submit this revised version of our manuscript entitled "Noise-Induced Reduction and Early Recovery of SPN OFF Responses". In addition, we thank the reviewers and the editors for their critical evaluation of our manuscript and for the constructive and encouraging feedback. We greatly appreciate the time and effort invested in assessing our work, as well as the positive acknowledgment of the additional analyses and revisions we implemented.

We addressed the remaining minor comments and made the corresponding changes in the manuscript. We are convinced that these revisions have further strengthened the clarity and quality of the paper.

The final changes made to the manuscript are:

1. The abstract figure has been finalized and its figure legend added.
2. As requested, panels 6 D&E were removed. Instead, we added a raw data set for IPSC amplitudes at the 24hrs time point to visualize the change in amplitudes following noise exposure.
3. All figure legends now state number of cells and animals. Figures only represent data for animals, not cells. The use of bar graphs (mean ±SD) vs. box plots (median, IQR, percentiles) are clearly stated in the methods.
4. Throughout the text, remaining typos and inconsistencies (e.g. 24-hr vs 24hrs) were corrected.

Yours sincerely,

C. Kopp-Scheinpflug

Conny Kopp-Scheinpflug

2nd Revision - Editorial Decision 25-Mar-2026

Dear Dr Kopp-Scheinpflug,

Re: JP-RP-2026-289987R2 "**Noise-Induced Reduction and Early Recovery of SPN OFF Responses**" by Mihai Stancu, Ezhilarasan Rajaram, Joseph Kroeger, Benedikt Grothe, and Conny Kopp-Scheinpflug

Thank you for submitting your manuscript to The Journal of Physiology. It has been assessed by a Reviewing Editor and we are pleased to tell you that it is acceptable for publication following satisfactory minor revision.

The referee reports are copied at the end of this email.

Please address all the points raised and incorporate all requested revisions or explain in your Response to Referees why a change has not been made. We hope you will find the comments helpful and that you will be able to return your revised manuscript within 4 weeks. If you require longer than this, please contact journal staff: jp@physoc.org.

Your revised manuscript should be submitted online using the link in your Author Tasks Link Not Available. This link is accessible via your account as Corresponding Author; it is not available to your co-authors. If this presents a problem, please contact journal staff (jp@physoc.org). Image files from the previous version are retained on the system. Please ensure you replace or remove any files that are being revised.

If you do not wish to submit a revised version of your manuscript, you must inform our journal staff (jp@physoc.org) or reply to this email to request withdrawal. Please note that a manuscript must be formally withdrawn from the peer review process at one journal before it may be submitted to another journal.

TRANSPARENT PEER REVIEW POLICY: To improve the transparency of its peer review process The Journal of Physiology publishes online, as supporting information, the peer review history of all articles accepted for publication. Readers will have access to decision letters, including Editors' comments and referee reports, for each version of the manuscript, as well as any author responses to peer review comments. Referees can decide whether or not they wish to be named on the peer review history document.

ABSTRACT FIGURES: Authors are expected to use The Journal's premium BioRender account to create/redraw their Abstract Figures. Information on how to access this account is here: https://physoc.onlinelibrary.wiley.com/journal/14697793/biorender-access.

This will enable Authors to create and download high-resolution figures. If authors have used the free BioRender service, they can use the instructions provided in the link above to download a high-resolution version suitable for publication.

The link provided should only be used for the purposes of this submission. Authors will be charged for figures created on this account if they are not related to this manuscript submission.

LANGUAGE EDITING AND SUPPORT FOR PUBLICATION: If you would like help with English language editing, or other article preparation support, Wiley Editing Services offers expert help, including English Language Editing, as well as translation, manuscript formatting, and figure formatting at www.wileyauthors.com/eeo/preparation. You can also find resources for Preparing Your Article for general guidance about writing and preparing your manuscript at www.wileyauthors.com/eeo/prepresources.

RIGOUR AND REPRODUCIBILITY: We recommend authors consult The Journal's Rigour and Reproducibility requirements to ensure that their manuscript is suitable for publication: https://physoc.onlinelibrary.wiley.com/pb-assets/hub-assets/physoc/documents/TJP-Rigour-and-Reproducibility-Requirements.pdf

REVISION CHECKLIST:

Check that your Methods section conforms to journal policy: https://jp.msubmit.net/cgi-bin/main.plex?form_type=display_requirements#methods

Check that data presented conforms to the statistics policy: https://jp.msubmit.net/cgi-bin/main.plex?form_type=display_requirements#statistics

Upload a full Response to Referees file. To create your 'Response to Referees' copy all the reports, including any comments from the Senior and Reviewing Editors, into a Microsoft Word, or similar, file and respond to each point, using font or background colour to distinguish comments and responses and upload as the required file type.

Please upload two versions of your manuscript text: one with all relevant changes highlighted and one clean version with no changes tracked. The manuscript file should include all tables and figure legends, but each figure/graph should be uploaded as separate, high-resolution files. The journal is now integrated with Wiley's Image Checking service. For further details, see: https://www.wiley.com/en-us/network/publishing/research-publishing/trending-stories/upholding-image-integrity-wileys-image-screening-service

You may also upload:

- 'Potential Cover Art' for consideration as the issue's cover image
- Appropriate Supporting Information (Video, audio or data set: see https://jp.msubmit.net/cgi-bin/main.plex?form_type=display_requirements#supp).

We look forward to receiving your revised submission.

If you have any queries, please reply to this email and we will be pleased to advise.

Best wishes,

Vaughan Macefield
Senior Editor
The Journal of Physiology

REQUIRED ITEMS

- Your manuscript must include a complete Additional Information section, including competing interests; funding; author contributions and acknowledgements.

EDITOR COMMENTS

Reviewing Editor:

Dear Authors,

Thank you for carefully addressing the remaining minor concerns.

Please see 'Required Items' above.

Senior Editor:

Thank you for submitting your revised manuscript to The Journal of Physiology. While the Reviewer Editor and I are satisfied with your amendments, before we can accept your paper you need to include the Additional Information section (acknowledgements, funding, author contributions, competing interests) within the text. I look forward to receiving the amended manuscript shortly.

END OF COMMENTS

LMU
LUDWIG-
MAXIMILIANS-
UNIVERSITÄT
MÜNCHEN

FACULTY OF BIOLOGY
NEUROBIOLOGY

Prof. Dr. C. Kopp-Scheinpflug · Faculty of Biology · Großhaderner Str. 2 · 82152 Planegg-Martinsried

The Journal of Physiology

Prof. Dr. C. Kopp-Scheinpflug

Telefon +49 (0)89 2180-74310

cks@bio.lmu.de

https://www.bio.lmu.de/en/research/research-fields/neurobiology/kopp-scheinpflug-lab/

Postanschrift:
Großhaderner Straße 2
D-82152 Planegg-Martinsried

Ihr Zeichen, Ihre Nachricht vom Unser Zeichen

Martinsried, 26.03.2026

The Journal of Physiology Decision on Our Manuscript: "**Noise-Induced Reduction and Early Recovery of SPN OFF Responses**", JP-RP-2026-289987R2.

Dear Editors,

my apologies – since all the additional information was provided on the JP platform, I was not aware I had to also include it into the manuscript file.

All the additional information is now provided in the manuscript file on page 19 between the figure legends and the references.

Thank you for your patience!!

Yours sincerely,

C. Kopp-Scheinpflug

Conny Kopp-Scheinpflug

Dear Professor Kopp-Scheinpflug,

Re: JP-RP-2026-289987R3 "**Noise-Induced Reduction and Early Recovery of SPN OFF Responses**" by Mihai Stancu, Ezhilarasan Rajaram, Joseph A. Kroeger, Benedikt Grothe, and Conny Kopp-Scheinpflug

We are pleased to tell you that your paper has been accepted for publication in The Journal of Physiology.

The last Word (or similar) version of the manuscript provided will be used by the Production Editor to prepare your proof. When this is ready you will receive an email containing a link to Wiley's Online Proofing System. The proof should be thoroughly checked and corrected as promptly as possible.

Authors should note that it is too late at this point to offer corrections prior to proofreading. Major corrections at proof stage, such as changes to figures, will be referred to the Editors for approval before they can be incorporated. Only minor changes, such as to style and consistency, should be made at proof stage. Changes that need to be made after proof stage will usually require a formal correction notice.

All queries at proof stage should be sent to: TJP@wiley.com.

Following copyediting and typesetting the accepted version will be published online.

Yours sincerely,

Vaughan Macefield
Senior Editor
The Journal of Physiology

IMPORTANT POINTS TO NOTE FOLLOWING ACCEPTANCE OF YOUR PAPER:

• IMPORTANT NOTICE ABOUT OPEN ACCESS: To assist authors whose funding agencies mandate immediate public access to published research findings, The Journal of Physiology allows authors to pay an Open Access (OA) fee to have their papers made freely available immediately on publication.

The Corresponding Author will receive an email from Wiley with details on how to register or log in to Wiley Authors where you will be able to place an order.

• You can check if your funder or institution has a Wiley Open Access Account here:
https://authors.wiley.com/author-resources/Journal-Authors/open-access/author-compliance-tool.html

• TRANSPARENT PEER REVIEW POLICY: To improve the transparency of its peer review process, The Journal of Physiology publishes online as supporting information the peer review history of all articles accepted for publication. Readers will have access to decision letters, including Editors' comments and referee reports, for each version of the manuscript as well as any author responses to peer review comments. Referees can decide whether or not they wish to be named on the peer review history document.

• You can help your research get the attention it deserves! Check out Wiley's free Promotion Guide for best-practice recommendations for promoting your work at: www.wileyauthors.com/eeo/guide. You can learn more about Wiley Editing Services which offers professional video, design, and writing services to create shareable video abstracts, infographics, conference posters, lay summaries, and research news stories for your research at: www.wileyauthors.com/eeo/promotion.

• If you would like to receive our 'Research Roundup', a monthly newsletter highlighting the cutting-edge research published in The Physiological Society's family of journals (The Journal of Physiology, Experimental Physiology, Physiological Reports, The Journal of Nutritional Physiology and The Journal of Precision Medicine: Health and Disease), please click this link, fill in your name and email address and select 'Research Roundup':
https://www.physoc.org/journals-and-media/membernews

EDITOR COMMENTS

Senior Editor:

Comments to the Author:

Thank you for attending to this remaining issue. I am pleased to report that your manuscript is now considered acceptable for publication in The Journal of Physiology.
